# Supplementary material for: Variant connective tissue (joint hypermobility) and its relevance to depression and anxiety in adolescents: a cohort-based case–control study
Source: BMJ Open. 2022 Nov 15;12(12):e066130. doi: 10.1136/bmjopen-2022-066130 (PMC9723902; doi:10.1136/bmjopen-2022-066130)
Supplement: Author's manuscript [file bmjopen-2022-066130.draft_revisions.pdf]

BMJ Open is committed to open peer review. As part of this commitment we make the peer review history of every article we publish publicly available.

When an article is published we post the peer reviewers' comments and the authors' responses online. We also post the versions of the paper that were used during peer review. These are the versions that the peer review comments apply to.

The versions of the paper that follow are the versions that were submitted during the peer review process. They are not the versions of record or the final published versions. They should not be cited or distributed as the published version of this manuscript.

BMJ Open is an open access journal and the full, final, typeset and author-corrected version of record of the manuscript is available on our site with no access controls, subscription charges or pay-per-view fees (http://bmjopen.bmj.com).

If you have any questions on BMJ Open's open peer review process please email info.bmjopen@bmj.com

BMJ Open

Variant connective tissue (joint hypermobility) and its relevance to depression and anxiety in adolescents: a cohort-based case-control study

Journal:	*BMJ Open*
Manuscript ID	bmjopen-2022-066130
Article Type:	Original research
Date Submitted by the Author:	30-Jun-2022

Complete List of Authors:	Eccles, Jessica; Brighton and Sussex Medical School, Department of Neuroscience; Sussex Partnership NHS Foundation Trust<br>Quadt, Lisa; Brighton and Sussex Medical School, Department of Neuroscience; Sussex Partnership NHS Foundation Trust<br>McCarthy, Hannah ; Brighton and Sussex Medical School, Department of Neuroscience; University Hospitals Dorset NHS Foundation Trust<br>Davies, Kevin; Brighton and Sussex Medical School<br>Bond, Rod ; University of Sussex, School of Psychology<br>David, Anthony; UCL, Institute of Mental Health<br>Harrison, Neil; Cardiff University, Brain Research Imaging Centre; Brighton and Sussex Medical School, Department of Neuroscience<br>Critchley, Hugo; Brighton and Sussex Medical School, Neuroscience; Sussex Partnership NHS Foundation Trust

Keywords:	MENTAL HEALTH, Paediatric rheumatology < PAEDIATRICS, PAIN MANAGEMENT, Anxiety disorders < PSYCHIATRY, Child & adolescent psychiatry < PSYCHIATRY, Depression & mood disorders < PSYCHIATRY

SCHOLARONE™
Manuscripts

I, the Submitting Author has the right to grant and does grant on behalf of all authors of the Work (as defined in the below author licence), an exclusive licence and/or a non-exclusive licence for contributions from authors who are: i) UK Crown employees; ii) where BMJ has agreed a CC-BY licence shall apply, and/or iii) in accordance with the terms applicable for US Federal Government officers or employees acting as part of their official duties; on a worldwide, perpetual, irrevocable, royalty-free basis to BMJ Publishing Group Ltd ("BMJ") its licensees and where the relevant Journal is co-owned by BMJ to the co-owners of the Journal, to publish the Work in this journal and any other BMJ products and to exploit all rights, as set out in our licence.

The Submitting Author accepts and understands that any supply made under these terms is made by BMJ to the Submitting Author unless you are acting as an employee on behalf of your employer or a postgraduate student of an affiliated institution which is paying any applicable article publishing charge ("APC") for Open Access articles. Where the Submitting Author wishes to make the Work available on an Open Access basis (and intends to pay the relevant APC), the terms of reuse of such Open Access shall be governed by a Creative Commons licence – details of these licences and which Creative Commons licence will apply to this Work are set out in our licence referred to above.

Other than as permitted in any relevant BMJ Author's Self Archiving Policies, I confirm this Work has not been accepted for publication elsewhere, is not being considered for publication elsewhere and does not duplicate material already published. I confirm all authors consent to publication of this Work and authorise the granting of this licence.

For peer review only

Variant connective tissue (joint hypermobility) and its relevance to depression and anxiety in adolescents: a cohort-based case-control study

Jessica A Eccles0000-0002-0062-1216, Lisa Quadt, Hannah E McCarthy, Kevin A Davies, Rod Bond, Anthony S David, Neil A Harrison, Hugo D Critchley

Department of Neuroscience, Brighton and Sussex Medical School, Trafford Centre, BN1 9RY, Brighton, UK
Jessica A Eccles, Clinical Senior Lecturer
Lisa Quadt, Research Fellow
Hannah E McCarthy, Specialty Registrar
Neil A Harrison, Honorary Consultant Professor of Neuropsychiatry
Hugo D Critchley, Head of Department

Sussex Partnership NHS Foundation Trust, Arundel Road, Worthing, BN1 2EP, UK
Jessica A Eccles
Lisa Quadt, Research Fellow
Hugo D Critchley

Royal Bournemouth and Christchurch Hospitals NHS Foundation Trust, Castle Lane East, Dorset, BH7 7DW, UK
Hannah E McCarthy, Specialty Registrar

University Hospitals Sussex NHS Trust, Lyndhurst Road, Worthing, BN11 2DH UK
Jessica A Eccles
Kevin A Davies, Emeritus Foundation Professor of Medicine

Department of Clinical and Experimental Medicine, Brighton and Sussex Medical School, BSMS Teaching Building, Brighton, BN1 9XP, UK
Kevin A Davies, Emeritus Foundation Professor of Medicine

School of Psychology, University of Sussex, Sussex House, Brighton, BN1 9RH, UK
Rod Bond, Emeritus Reader

UCL Institute for Mental Health, University College London, Gower Street, London, WC1E 6BT, UK
Anthony S David, Professor of Mental Health

Cardiff University Brain Research Imaging Centre, Cardiff, CF24 4HQ, UK
Neil A Harrison, Clinical Professor in Neuroimaging

*Correspondence to: Jessica A Eccles, Department of Neuroscience, Brighton and Sussex Medical School, Trafford Centre, University of Sussex, BRIGHTON, BN1 9RY, J.Eccles@bsms.ac.uk

For peer review only - http://bmjopen.bmj.com/site/about/guidelines.xhtml

Abstract

Objective: To test whether variant connective tissue structure, as indicated by the presence of joint hypermobility, poses a developmental risk for mood disorders in adolescence.

Design: Cohort-based case-control study.

Setting: Data from the Avon Longitudinal Study of Parents and Children (ALSPAC) were interrogated.

Participants: 6105 children of the ALSPAC cohort at age 14 years old, of whom 3803 also were assessed when aged 18 years.

Main outcome measures: In a risk analysis, we examined the relationship between generalised joint hypermobility (GJH) at age 14 years with psychiatric symptoms at age 18 years. In an association analysis, we examined the relationship between presence of symptomatic joint hypermobility syndrome (JHS) and ICD-10 diagnoses of depression and anxiety (CIS-R, ASI) at age 18 years.

Results: GJH was more common in females (n=856, 28%) compared to males (n=319, 11%; OR 3.20 (95% CI 2.78 to 3.68); P<0.001). In males only, GJH at age 14 years predicted depression at 18 years (OR 2.10 (95%CI 1.17 to 3.76)); an index of basal physiological arousal, elevated resting heart rate, mediated this effect. Across genders, the diagnosis of JHS at age 18 years was associated with the presence of depressive disorder (adjusted OR 3.52 (95%CI 1.67 to 7.40)), anxiety disorder (adjusted OR 3.14 (95% CI 1.52 to 6.46)), level of anxiety (B=8.08, $t(3278)=3.95$, P<0.001), and degree of psychiatric symptomatology (B=0.096, $t(2064)=4.38$).

Conclusions: Variant collagen, indexed by joint hypermobility, is linked to the emergence of depression and anxiety in adolescence, an effect mediated by autonomic factors in males. Recognition of this association may motivate further evaluation, screening, and interventions to mitigate development of psychiatric disorders and improve health outcomes.

Strengths and limitations of this study

- We used a birth-cohort in a longitudinal case-control study to investigate if hypermobility at age 14 was a predictor for psychiatric symptoms at age 18 in males and females.

For peer review only - http://bmjopen.bmj.com/site/about/guidelines.xhtml

- In a risk analysis, we tested for a relationship between Generalized Joint Hypermobility at age 14 years and depression and anxiety at age 18, and whether physiological arousal had a mediating role on this relationship.
- In an association analysis, we examined the effects of symptomatic Joint Hypermobility Syndrome on psychiatric symptoms in males and females at 18 years.
- Classification of (symptomatic) joint hypermobility is still heavily debated, with varying cut-offs and remaining questions about the Beighton Scale as the most widely used measurement tool.
- Contributions of variant connective tissue/joint hypermobility are likely complex and will require more systematic scientific inquiry.

For peer review only

Introduction

Adolescence is a critical time in psychological and physical development. An individual's trajectory from child into adult is influenced by interacting biological, psychological, environmental, social and cultural factors that determine the subsequent life journey. One quarter of the world's population is aged between 10 and 24 years, and around 75% of psychiatric disorders emerge during adolescence with most fully expressed before age 25 years. Psychiatric disorders in young people now represent half of the global burden of disease, and frequently carry poor long-term outcomes. [1] Longitudinal studies can provide valuable insight into the development of psychiatric disorders. [2] Furthermore, identification of risk factors can inform preventative and early interventional strategies to promote well-being and reduce future morbidity. [3 4]

Generalized joint hypermobility (GJH) is a characteristic marker of hereditary disorders of connective tissue, compromising a matrix of proteins that includes collagens, elastins, fibrillins, and tenascins. [5] Joint hypermobility is typically assessed using the Beighton scale, [6] and is more common in females, declining with age. GJH itself is not necessarily a medical problem, but clinical phenotypes including hypermobile EDS (hEDS; formerly known as EDS hypermobility type/EDS type-III/Joint hypermobility syndrome; JHS) [7], and hypermobility spectrum disorder[8] (including generalized joint hypermobility; GJH) are associated with clinically significant issues. General population estimates show that approximately 20% of adolescents have GJH. [9] GJH also is associated with psychiatric conditions and psychological symptoms, [10] where adults with GJH are over-represented among patients expressing common mental disorders; i.e. depression, anxiety and panic. [11] Correspondingly, neuroimaging studies of individuals with joint hypermobility have identified structural and functional differences within brain regions implicated in emotional arousal, reactivity and feelings, notably amygdala[12] and insula. [13] Joint hypermobility is also linked to autonomic problems (dysautonomia), [14] often expressed as postural tachycardia syndrome (PoTS), a condition characterized by accelerated heart rate on standing, and other symptoms that overlap phenomenologically with anxiety. [15] Adolescents with PoTS have greater resting heart rates than those without PoTS. [16] Nevertheless, in children and adolescents, there remains little information about how joint hypermobility might be associated with common psychiatric conditions. [17]

The objective of this study was to test, for the first time longitudinally, whether there is an association between variant collagen, indexed by joint hypermobility (GJH and JHS)

For peer review only - http://bmjopen.bmj.com/site/about/guidelines.xhtml

identified in early adolescence, and subsequent risk of depression and anxiety in later adolescence using a population-based birth cohort. We also sought to quantify this relationship and identify potential mediating factors.

Methods

Study design and participants

In this cohort-based case-control study, we used data from the The Avon Longitudinal Study of Parents and Children (ALSPAC) birth cohort to establish risk of psychiatric disorders for children and young people with and without GJH. The ALSPAC birth cohort follows children of 14062 live births from women living in (the former) Avon County, a geographically defined region in southwest England, with expected dates of delivery between April 1991 and December 1992 (http://www.bristol.ac.uk/alspac).[18] Parents completed regular postal questionnaires about all aspects of their child's health and development from birth. From the age of 7 years, children attended annual assessment clinics, during which they underwent a variety of biomedical and physical tests, face-to-face interviews and standardized assessments of mental health. Data collection followed written informed consent.

Analyses for our study were based on two samples: One sample was used for a predictor model, and the second for an association model (see Fig. 1). The 'risk-set' for the predictor model included 6105 adolescents who underwent a complete joint hypermobility assessment in a clinical setting as 14 years old adolescents. Of this risk set, 5731 adolescents also had heart rate data recorded at the same time-point. Of these, 3803 adolescents completed assessments for depression and anxiety (CIS-R) aged 18 years. The second (association) sample included the 4390 adolescents who completed assessments for both joint hypermobility and the CIS-R aged 18 years. Finally, data from a subset of 3443 adolescents from the association sample, who also completed an assessment for chronic widespread pain at the same 18-year time point, were additionally analyzed.

Outcomes

ALSPAC participants underwent measurement of Generalized joint hypermobility (GJH) in a dedicated research clinic, by trained assessors using the modified Beighton 9-point scoring system[6] at two time points; age 14 years and age 18 years. Each joint was assessed separately. The fifth metacarpophalangeal joint was scored as hypermobile if it could be extended >90°, the thumb if it could be opposed to the wrist, and the elbows and knees if they could be

For peer review only - http://bmjopen.bmj.com/site/about/guidelines.xhtml

extended >10°. The spine was scored as hypermobile if both palms could be placed flat on the floor with the knees straight. Scores were recorded for individual joints, and a total score (maximum of 9) was ascertained. In common with other investigators, we used a cut-off point of ≥4 hypermobile joints as a dichotomized definition of the presence of GJH. [9] Resting heart rate was measured in the same assessment clinic setting, using a POLAR heart rate watch. A 'joint hypermobility syndrome' (JHS) variable was derived to determine if a participant had clinically significant hypermobility at age 18 years. JHS diagnosis is typically based on the revised Brighton criteria, [19] and requires a combination of major and minor criteria, comprising GJH in combination with significant musculoskeletal and/or connective tissue symptoms. Not all minor criteria were recorded in the ALSPAC cohort at age 18 years, thus JHS was operationalized as meeting two major criteria: the presence of GJH and concomitant chronic widespread pain (CWP) [20].

Depression and anxiety were measured at age 18 years, using the self-administered computerized version of the Clinical Interview Schedule-Revised (CIS-R). CIS-R is a standardized tool for measuring depression and anxiety in community samples, [21] including this cohort. The CIS-R quantifies symptoms to assign International Classification of Diseases-10 (ICD-10) diagnoses of depression and anxiety disorders. [22 23] We derived binary variables for; 1) the presence or absence of a primary diagnosis of major depression, and; 2) presence or absence of a primary diagnosis of any anxiety disorder (including generalized anxiety disorder, panic disorder, agoraphobia, social phobia and specific phobia). In addition, we derived two severity indices; 3) degree of psychiatric symptomatology (i.e., total CIS-R score), and 4) degree of anxiety from the Anxiety Sensitivity Index (total of 'mental and physical concerns' subscale).

Statistical Analyses

Statistical analyses were conducted in IBM SPSS Statistics Version 26 and MPlus Version 8. [24] We tested the hypothesis that joint hypermobility was a predictor for subsequent depression or anxiety at age 18 years, by first using logistic regression models to calculate the odds ratios (ORs) and 95% confidence intervals (CI) for depressive disorder and/or anxiety disorder at age 18 years, separately for individuals with and without GJH at age 14 years. Since joint hypermobility is more common in females, analyses for males and females were performed separately. To explore possible associations with dysautonomia (PoTS), we conducted separate logistic regression models to test the effect of resting heart rate (as an

For peer review only - http://bmjopen.bmj.com/site/about/guidelines.xhtml

index of physiological arousal) at age 14 years, on both GJH at aged 14 years and on the later expression of depression and anxiety at age 18 years.

We then performed a mediation analysis[25] with presence of depression at age 18 years as the binary outcome variable, GJH at age 14 years as the predictor, and heart rate at age 14 years as a potential mediating variable. We used probit regression with full information maximum likelihood (MPlus estimator command *ML* with Monte Carlo integration). Bootstrapping (n=2000) was applied, with the generation of 95% bias-corrected bootstrap confidence intervals to test inferentially for direct and indirect mediation effects. Analyses were performed separately for males and females. Mediation analyses were controlled for mean-centered age at assessment and body mass index, as both were associated with the potential mediator (heart rate).

In order to test for an association between symptomatic hypermobility (JHS) and concurrent psychiatric disorders, separate logistic regression models were used to calculate ORs and 95% CI for depression and anxiety disorder at age 18 years in adolescents with and without JHS. Linear regression was used to test for differences between individuals with and without JHS in total CIS-R score and anxiety sensitivity. As data were collected at the same time point, these analyses were not subject to maximum likelihood estimation.

For our principle longitudinal analyses, we applied full information maximum likelihood estimation (FIML), [26] where a likelihood function calculates the relationship between a probability estimate based on observed data from variables in the respective analytical model and different estimate values. FIML chooses parameter estimates that maximize this likelihood function based on complete cases, thereby providing robust parameter estimates, despite missing data. [27]

Patient and Public Involvement

This research was motivated by ongoing Patient and Public Involvement (PPI) engagement, in which concerns were regularly expressed by parents of young people with joint hypermobility. PPI members often felt dismissed by medical professionals, and regularly expressed a general need to enhance awareness of joint hypermobility as a risk factor for their children. The underappreciation of its complexity and associated risks were described as barriers to effective care. Our PPI members will help with disseminating our findings among patient groups in an accessible manner.

For peer review only - http://bmjopen.bmj.com/site/about/guidelines.xhtml

Results

Participants

Participant characteristics in the two samples at assessment time points are displayed in Table 1, and participant flow is displayed in Figure 1.

Table 1: Participant Characteristics in the two study samples

Timepoint	Evaluation	Participants with available data (n, % male)	Age (mean±SD, range)
**Risk Sample**			
Clinic aged 14	Generalized Joint Hypermobility<br>Heart rate	6105 (49% male)	13·8±0·2 yrs, 12·5-15·2
Clinic aged 18	Depression<br>Anxiety	3803 (45% male)	17·7±0·4 yrs, 16·3-19·3
**Association Sample**			
Clinic aged 18	Joint Hypermobility Syndrome<br>Depression<br>Anxiety	3557 (42% male)	17·7±0·4 yrs, 16·3-19·3

Prevalence of hypermobility and association with age and sex assigned at birth

Of those assessed at 14 years, 1175 (19%) had GJH as reported elsewhere.[9] GJH was more common in females (n=856, 28%) compared to males (n=319, 11%; OR 3.20 [95% CI 2.78-3.68]; p<0.001) and not associated with variation in age.

Of those who had assessments for joint hypermobility and chronic widespread pain (CWP) at 18 years, 36 (1%) had both GJH and CWP, and thus met operationalized criteria for JHS. This was significantly more common in females; only 3 were male (OR 8.04 [95% CI 2.46-26.25]; p=0.001). JHS was not associated with variation in age.

Risk set analysis

Risk of depression and anxiety at age 18 years according to hypermobility status at age 14 years

Table 2 displays the proportion of participants with GJH at age 14 years in whom depression or anxiety was indexed at age 18 years. Across the whole group, GJH was a significant predictor of depression at 18 (OR 1.70 [95% CI 1.31-2.22]; p=0.001), where a greater proportion of individuals with depression were hypermobile (had GJH) than those without depression (Table 2, Figure 2A). Additionally, we observed a significant interaction of JH and sex on depression ($F_{(2,3802)}$=3.73, p=0.024), where this effect was significant only in males (OR 2.10 [95% CI 1.17-3.76]; p=0.013; Table 2, Figure 2B).

In this analysis, GJH at 14 years was not associated with anxiety at 18 years.

For peer review only - http://bmjopen.bmj.com/site/about/guidelines.xhtml

Table 2: Relationship between hypermobility at age 14 years and depression and anxiety aged 18 years

	Whole group	Male	Female
**No ICD-10 Depression**			
Patients with available data (N)	3510	1627	1883
Proportion of GJH (n, %, 95% CI)	688 (19·6%, 18·31-20·96)	166 (10·2%, 8·82-11·77)	522 (27·74%, 25·76-29·81)
**ICD-10 Depression**			
Patients with available data (N)	293	78	215
Proportion of GJH (n, %, 95% CI)	86 (29·35%, 24·42-34·48)	15 (19·23%, 12·01-29·33)	71 (33·02%, 27·08-39·56)
**No ICD-Anxiety**			
Patients with available data (N)	3437	1604	1832
Proportion of GJH (n, %, 95% CI)	691 (20·10%, 18·79-21·47)	168 (10·47%, 9·06-12·06)	523 (28·55%, 26·53-30·66)
**ICD-10 Anxiety**			
Patients with available data (N)	366	101	265
Proportion of GJH (n, %, 95% CI)	83 (22·68%, 16·69-27·24)	13 (12·87%, 7·68-20·78)	70 (26·41%, 21·48-32·04)

Associations with heart rate at age 14 years

Heart rate was significantly higher in females (M=83.92, SD=11.94) than males (M=78.6, SD=11.44; t(5725)=17.18, p<0.001), and was significantly associated with variations in exact age (months) (r(5729)=-0.32, p=0.016) and BMI (r(5722)=0.081, p<0.001) at assessment. After correcting for age and BMI, heart rate at 14 years was significantly associated with both GJH at age 14 years (OR 1.01 [95% CI 1.01-1.02]; p<0.001), and also with depression at 18 years (OR=1.01 [95% CI 1.00-1.02], p=0.007).

Risk of depression at age 18 years

The OR of depression at 18 years in both males and females, given GJH at 14 years, was 1.28 (b=0.246 [95% CI 1.12-1.47, p<0.001). In males, the OR was 1.38 (b=0.032 [95% CI 0.97 to 1.79] p=0.036). There was no significant effect in females only.

Mediation analysis

A mediation analysis was only conducted in males, for whom the relationship between GJH and depression was significant. Both GJH and heart rate (at age 14 years) were significantly associated with depression (at age 18 years). The mediation analysis showed that the relationship between GJH and depression was mediated by heart rate (Fig. 3). This was demonstrated by the bootstrap confidence interval derived from 2000 samples of the indirect effect of heart rate on the relationship between GJH and depression being significant (b=1.05 [95% CI 1.011-1.125]) when controlling for mean centered age and BMI.

For peer review only - http://bmjopen.bmj.com/site/about/guidelines.xhtml

Association set analysis

Symptomatic hypermobility (JHS) and depression at age 18 years

A higher proportion of JHS was found in participants who met ICD-10 criteria for depression (Fig. 4A). Across the whole group, JHS was significantly associated with depression at age 18 years (Fig. 5 shows ORs for this effect, which remained significant when adjusted for sex). Given the low levels of JHS in males (n=3), this analysis was not powered to be undertaken separately but remained significant for females (Fig. 5).

Across the whole group, higher CIS-R scores were present in the JHS group, compared to those without JHS (B=5.89, $t(3442)=5.50$, $p<0.001$; Fig. 6A). In analyses corrected for sex, this association remained (B=4.97, $t(3441)=4.73$, $p<0.001$). In females, higher CIS-R scores were present in those with JHS (M=11.97, SD=10.13), compared to those without JHS (M=6.61, SD=6.88) (B=5.36, $t(1992)=4.40$, $p<0.001$).

Symptomatic hypermobility (JHS) and anxiety at age 18

The prevalence of JHS was greater among participants who met ICD-10 criteria for an anxiety disorder (Fig. 4B). JHS was significantly associated with any anxiety disorder at age 18 years, a relationship that remained significant when adjusted for sex. ORs are displayed in Figure 5.

Across the whole group, an elevated anxiety sensitivity score was observed in individuals with JHS, compared to those without JHS (B=8.08, $t(3278)=3.95$, $p<0.001$; Fig. 6B). This association remained after adjusting for sex (B=6.60, $t(3278)=3.28$, $p=0.001$). In females, higher anxiety sensitivity was present in those with JHS (M=55.94, SD=12.26), compared to those without JHS (M=49.33, SD=11.76).

Discussion

In adults, the link between variant connective tissue structure (indexed by joint hypermobility) and affective psychopathology is largely established[11] and, interestingly, reinforced by convergent evidence from domestic dogs. [28] Our results extend these findings by showing a link between GJH at age 14 years and subsequent depression in adolescence. There was a significant interaction of sex with significant effects in males only. We also show a mediating effect of basal autonomic physiology (indexed by heart rate) on this relationship. However, we found no longitudinal association between GJH alone and anxiety. This observation is striking as such an association is widely reported in adults. [10 11 29] However, symptomatic joint hypermobility (JHS; operationalized here as the presence of

For peer review only - http://bmjopen.bmj.com/site/about/guidelines.xhtml

GJH and chronic widespread pain) predicted both anxiety and depression in female adolescents and across the whole group when adjusted for sex.

Variant connective tissue, expressed as joint hypermobility, is characteristically a familial trait. However, no single gene is implicated with consistency. Nevertheless, symptomatic hypermobility (hEDS /HSD/JHS) is considered to be an autosomal dominant trait with incomplete penetrance, variable expressivity, and influenced by sex, since joint hypermobility is more common in females. [30] However, despite this female preponderance, joint hypermobility is also associated with neurodivergence[31] (including Fragile X syndrome, Attention Deficit Hyperactivity Disorder and Autism), which is more commonly diagnosed in males. Importantly, however reported co-occurrences are potentially skewed by underdiagnosis of neurodivergence in females. [32] Neurodivergence also increases the likelihood of clinical anxiety and depression, [33] and it is plausible that this mechanism may account for the observed association between GJH at 14 years and high rates of depression at age 18 years in males.

Hypermobility is associated with dysautonomia, [34] typically postural tachycardia syndrome (PoTS), symptoms of which have phenomenological overlap with anxiety. [15] Adolescents with PoTS have greater resting heart rates than peers without PoTS. [16] Our present finding of a mediating role of heart rate on the association between hypermobility and depression may thus indicate the presence of dysautonomia and constitutional differences in physiological reactivity. Interestingly, a Swedish cohort study also demonstrated that adolescent males with higher basal heart rate are more likely to develop subsequent psychopathology, notably anxiety disorders. [35] However, it should be noted that the size of effect linking heart rate with hypermobility and depression was small.

Our data suggest that depression can be predicted, in males, by the presence of GJH. However, in females, additional features of symptomatic hypermobility (JHS), notably chronic widespread pain, are required. One possible explanation for this pattern of results is that the higher rates of anxiety and depression in females reflect susceptibility and perhaps exposure to a wider range of risk factors, which obscures an association with GJH apparent in males.

The categorization and classification of joint hypermobility and symptomatic joint hypermobility remains the subject of substantial debate. [8] Although the Beighton Scale remains the most widely-used approach for measuring hypermobility, there remain considerable questions about its utility, particularly in children and adolescents, [36] and cut-offs vary widely.

For peer review only - http://bmjopen.bmj.com/site/about/guidelines.xhtml

Our study provides evidence for both a longitudinal association between a common variant of connective tissue, hypermobility, and a common psychiatric illness, depression, at age 18 years. We also observed the more direct association between symptomatic joint hypermobility (JHS) and depression and anxiety at age 18 years. A more fine-grained assessment of mental health symptoms might have uncovered a broader range of outcomes. Recent years have seen increasing attention in the lay media to joint hypermobility as a single biomedical cause of sometimes profound disability. However, the contribution of underlying variant connective tissue structure is likely to be complex and involves interacting autonomic, psychological and physiological factors. Further systematic scientific inquiry is required. Together, our findings highlight the importance of a broad biopsychosocial approach to these complex conditions, highlighting the interplay between physical and mental health, with the possibility of screening for hypermobility in adolescence, the judicious use of which may provide opportunities for early intervention to identify and alleviate common psychiatric symptoms.

For peer review only - http://bmjopen.bmj.com/site/about/guidelines.xhtml

Acknowledgments

We are extremely grateful to all the families who took part in this study, the midwives for their help in recruiting them, and the whole ALSPAC team, which includes interviewers, computer and laboratory technicians, clerical workers, research scientists, volunteers, managers, receptionists and nurses.

Funding

Funding for this project came via a fellowship to JAE (MRC MR/K002643/1).

Author contributions

JAE acquired funding, conceptualized and designed the study, analysed and managed the data, and drafted this manuscript. LQ visualized data and revised the manuscript. RB and HS supported data analysis. All authors critically reviewed the manuscript and approved the final version.

Declaration of Interests

The authors declare no interests.

Data Sharing

All data is available on the ALSPAC data dictionary and variable search tool (http://www.bristol.ac.uk/alspac/researchers/our-data/).

Ethical Approval

Approval for the original ALSPAC study was obtained from the ALSPAC Ethics and Law Committee (IRB00003312) and Local Research Ethics Committees.

For peer review only - http://bmjopen.bmj.com/site/about/guidelines.xhtml

References

1. Fusar-Poli P. Integrated Mental Health Services for the Developmental Period (0 to 25 Years): A Critical Review of the Evidence. *Front Psychiatry* 2019;10:355. doi: 10.3389/fpsyt.2019.00355 [published Online First: 2019/06/25]
2. Osimo EF, Stochl J, Zammit S, et al. Longitudinal population subgroups of CRP and risk of depression in the ALSPAC birth cohort. *Comprehensive psychiatry* 2020;96:152143. doi: https://doi.org/10.1016/j.comppsych.2019.152143
3. McGorry PD. Is early intervention in the major psychiatric disorders justified? Yes. *BMJ* 2008;337 doi: 10.1136/bmj.a695
4. Colizzi M, Lasalvia A, Ruggeri M. Prevention and early intervention in youth mental health: is it time for a multidisciplinary and trans-diagnostic model for care? *Int J Ment Health Syst* 2020;14:23. doi: 10.1186/s13033-020-00356-9 [published Online First: 2020/04/01]
5. Baeza-Velasco C, Pailhez G, Bulbena A, et al. Joint hypermobility and the heritable disorders of connective tissue: clinical and empirical evidence of links with psychiatry. *Gen Hosp Psychiatry* 2015;37(1):24-30. doi: 10.1016/j.genhosppsych.2014.10.002 S0163-8343(14)00264-3 [pii] [published Online First: 2014/12/03]
6. Beighton P, Solomon L, Soskolne CL. Articular mobility in an African population. *Annals of the rheumatic diseases* 1973;32(5):413-8.
7. Tinkle BT, Bird HA, Grahame R, et al. The lack of clinical distinction between the hypermobility type of Ehlers-Danlos syndrome and the joint hypermobility syndrome (a.k.a. hypermobility syndrome). *Am J Med Genet A* 2009;149A(11):2368-70. doi: 10.1002/ajmg.a.33070 [published Online First: 2009/10/21]
8. Castori M, Tinkle B, Levy H, et al. A framework for the classification of joint hypermobility and related conditions. *Am J Med Genet C Semin Med Genet* 2017;175(1):148-57. doi: 10.1002/ajmg.c.31539 [published Online First: 2017/02/02]
9. Clinch J, Deere K, Sayers A, et al. Epidemiology of generalized joint laxity (hypermobility) in fourteen-year-old children from the UK: a population-based evaluation. *Arthritis and rheumatism* 2011;63(9):2819-27. doi: 10.1002/art.30435 [published Online First: 2011/05/07]
10. Sharp HEC, Critchley HD, Eccles JA. Connecting brain and body: Transdiagnostic relevance of connective tissue variants to neuropsychiatric symptom expression. *World J Psychiatry* 2021;11(10):805-20. doi: 10.5498/wjp.v11.i10.805 [published Online First: 2021/11/05]
11. Smith TO, Easton V, Bacon H, et al. The relationship between benign joint hypermobility syndrome and psychological distress: a systematic review and meta-analysis. *Rheumatology (Oxford)* 2014;53(1):114-22. doi: 10.1093/rheumatology/ket317 ket317 [pii] [published Online First: 2013/10/02]
12. Eccles JA, Beacher FD, Gray MA, et al. Brain structure and joint hypermobility: relevance to the expression of psychiatric symptoms. *Br J Psychiatry* 2012;200(6):508-9. doi: 10.1192/bjp.bp.111.092460 [published Online First: 2012/04/26]
13. Mallorqui-Bague N, Garfinkel SN, Engels M, et al. Neuroimaging and psychophysiological investigation of the link between anxiety, enhanced affective reactivity and interoception in people with joint hypermobility. *Front Psychol* 2014;5:1162. doi: 10.3389/fpsyg.2014.01162 [published Online First: 2014/10/30]
14. Celletti C, Camerota F, Castori M, et al. Orthostatic Intolerance and Postural Orthostatic Tachycardia Syndrome in Joint Hypermobility Syndrome/Ehlers-Danlos Syndrome, Hypermobility Type: Neurovegetative Dysregulation or Autonomic Failure? *BioMed research international* 2017;2017:9161865. doi: 10.1155/2017/9161865 [published Online First: 2017/03/14]
15. Mathias CJ, Low DA, Iodice V, et al. Postural tachycardia syndrome--current experience and concepts. *Nat Rev Neurol* 2012;8(1):22-34. doi: 10.1038/nrneurol.2011.187

For peer review only - http://bmjopen.bmj.com/site/about/guidelines.xhtml

nrneurol.2011.187 [pii] [published Online First: 2011/12/07]
16. Singer W, Sletten DM, Opfer-Gehrking TL, et al. Postural tachycardia in children and adolescents: what is abnormal? *The Journal of pediatrics* 2012;160(2):222-6. doi: 10.1016/j.jpeds.2011.08.054
17. Ezpeleta L, Navarro JB, Osa N, et al. Joint Hypermobility Classes in 9-Year-Old Children from the General Population and Anxiety Symptoms. *J Dev Behav Pediatr* 2018;39(6):481-88. doi: 10.1097/DBP.0000000000000577 [published Online First: 2018/05/31]
18. Boyd A, Golding J, Macleod J, et al. Cohort Profile: the 'children of the 90s'--the index offspring of the Avon Longitudinal Study of Parents and Children. *Int J Epidemiol* 2013;42(1):111-27. doi: 10.1093/ije/dys064 [published Online First: 2012/04/18]
19. Grahame R, Bird HA, Child A. The revised (Brighton 1998) criteria for the diagnosis of benign joint hypermobility syndrome (BJHS). *J Rheumatol* 2000;27(7):1777-9.
20. Deere KC, Clinch J, Holliday K, et al. Obesity is a risk factor for musculoskeletal pain in adolescents: findings from a population-based cohort. *Pain* 2012;153(9):1932-8. doi: 10.1016/j.pain.2012.06.006
S0304-3959(12)00360-0 [pii] [published Online First: 2012/07/19]
21. Lewis G, Pelosi AJ, Araya R, et al. Measuring psychiatric disorder in the community: a standardized assessment for use by lay interviewers. *Psychol Med* 1992;22(2):465-86. [published Online First: 1992/05/01]
22. Lewis G, Araya R. Classification, disability and the public health agenda. *Br Med Bull* 2001;57:3-15. [published Online First: 2001/11/27]
23. Brugha TS, Bebbington PE, Jenkins R, et al. Cross validation of a general population survey diagnostic interview: a comparison of CIS-R with SCAN ICD-10 diagnostic categories. *Psychol Med* 1999;29(5):1029-42. [published Online First: 1999/11/27]
24. Muthén L, Muthén B. Statistical Analysis with Latent Variables: Mplus User's Guide: Muthén & Muthén, Los Angeles, CA, 2012.
25. Muthen B, Asparouhov T. Causal Effects in Mediation Modeling: An Introduction With Applications to Latent Variables. *Struct Equ Modeling* 2015;22(1):12-23. doi: 10.1080/10705511.2014.935843
26. Enders CK. Applied missing data analysis. New York, NY, US: Guilford Press 2010.
27. Newman DA. Missing Data: Five Practical Guidelines. *Organizational Research Methods* 2014;17(4):372-411. doi: 10.1177/1094428114548590
28. Bowen J, Fatjo J, Serpell JA, et al. First evidence for an association between joint hypermobility and excitability in a non-human species, the domestic dog. *Sci Rep* 2019;9(1):8629. doi: 10.1038/s41598-019-45096-0 [published Online First: 2019/06/15]
29. Bulbena A, Gago J, Pailhez G, et al. Joint hypermobility syndrome is a risk factor trait for anxiety disorders: a 15-year follow-up cohort study. *Gen Hosp Psychiatry* 2011;33(4):363-70. doi: 10.1016/j.genhosppsych.2011.03.004
30. Castori M. Ehlers-danlos syndrome, hypermobility type: an underdiagnosed hereditary connective tissue disorder with mucocutaneous, articular, and systemic manifestations. *ISRN Dermatol* 2012;2012:751768. doi: 10.5402/2012/751768
31. Csecs JLL, Iodice V, Rae CL, et al. Joint Hypermobility Links Neurodivergence to Dysautonomia and Pain. *Frontiers in Psychiatry* 2022;12 doi: 10.3389/fpsyt.2021.786916
32. Allely CS. Understanding and recognising the female phenotype of autism spectrum disorder and the “camouflage” hypothesis: a systematic PRISMA review. *Advances in Autism* 2019;5(1):14-37. doi: 10.1108/aia-09-2018-0036
33. Katzman MA, Bilkey TS, Chokka PR, et al. Adult ADHD and comorbid disorders: clinical implications of a dimensional approach. *BMC Psychiatry* 2017;17(1):302. doi: 10.1186/s12888-017-1463-3
34. Gazit Y, Nahir AM, Grahame R, et al. Dysautonomia in the joint hypermobility syndrome. *Am J Med* 2003;115(1):33-40. doi: S0002934303002353 [pii] [published Online First: 2003/07/18]

For peer review only - http://bmjopen.bmj.com/site/about/guidelines.xhtml

35. Latvala A, Kuja-Halkola R, Ruck C, et al. Association of Resting Heart Rate and Blood Pressure in Late Adolescence With Subsequent Mental Disorders: A Longitudinal Population Study of More Than 1 Million Men in Sweden. *JAMA Psychiatry* 2016;73(12):1268-75. doi: 10.1001/jamapsychiatry.2016.2717
36. Simmonds J. Generalized joint hypermobility: a timely population study and proposal for Beighton cut-offs: Beighton cut-offs for generalized joint hypermobility. *Rheumatology (Oxford)* 2017 doi: 10.1093/rheumatology/kex190

For peer review only

For peer review only - http://bmjopen.bmj.com/site/about/guidelines.xhtml

Figure Legends

Figure 1: Flow diagram of participants in study samples

Figure 2: Prevalence of Generalized Joint Hypermobility.

Figure 2A shows the difference in prevalence of GJH in participants who meet diagnostic criteria for ICD-10 depression (blue bar) and those who do not (red bar). Figure 2B shows a significant difference in prevalence of GJH in depressed and non-depressed participants in male but not female participants. Error bars show 95% CI.

Figure 3: Heart rate mediates the relationship between GJH and depression

This figure displays standardized regression coefficients for the relationship between Generalized Joint Hypermobility and depression as mediated by heart rate (solid black arrows), with the direct (dashed black arrow) and significant indirect effects (solid black arrow). Effects of covariates on heart rate and depression are displayed with grey dashed arrows.

Figure 4: Prevalence of Joint Hypermobility Syndrome

This figure shows the difference in prevalence of JHS in participants who meet ICD-10 criteria (blue bars) for depression (4A), and anxiety (4B), and those who do not meet diagnostic criteria (red bars).

Figure 5: Forest plot of odds ratios

This figure displays the odds rations and their 95% CIs of depression and anxiety given the presence of Joint Hypermobility Syndrome in the whole group, adjusted for sex, and in females only.

Figure 6: Mean CIS-R scores and Anxiety Sensitivity Index scores

Figure 6A displays the group difference in mean CIS-R (depression) score between participants with (blue dot) and without (red dot) JHS. Figure 6B displays the group difference in mean Anxiety Sensitivity Index score between participants with (blue dot) and without (red dot) JHS. Error bars shows +/- 1 SEM.

For peer review only

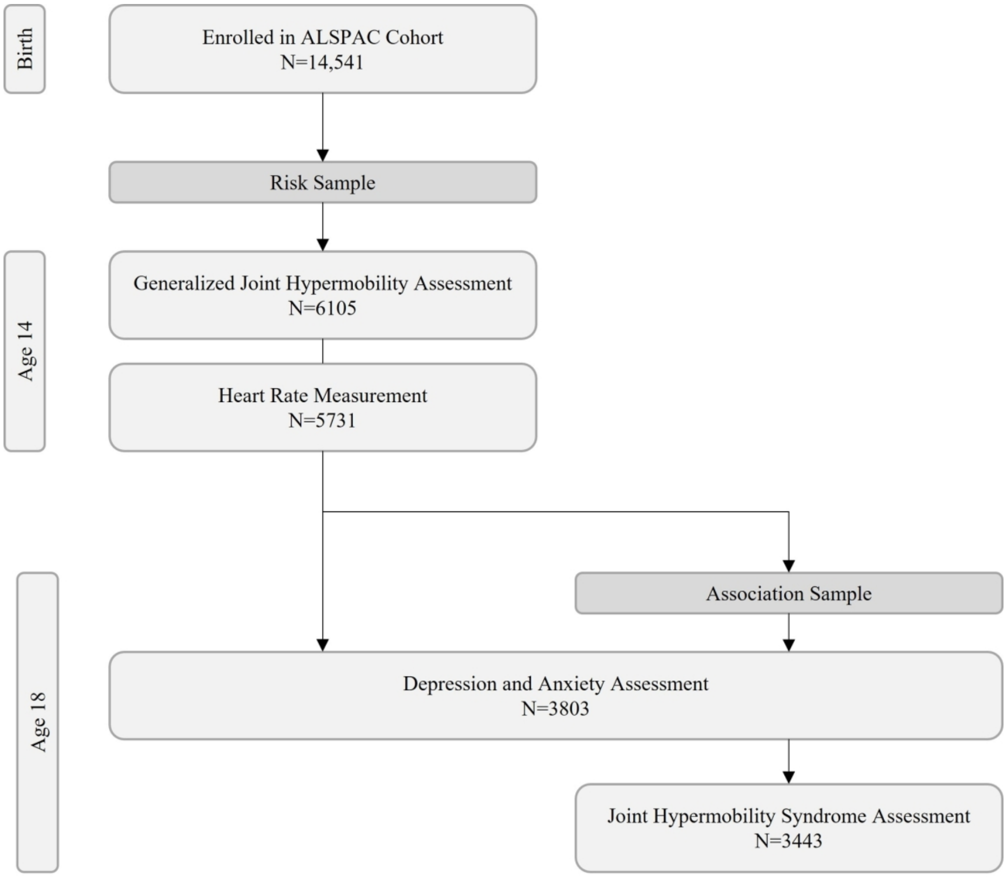

Figure 1: Flow diagram of participants in study samples

169x162mm (300 x 300 DPI)

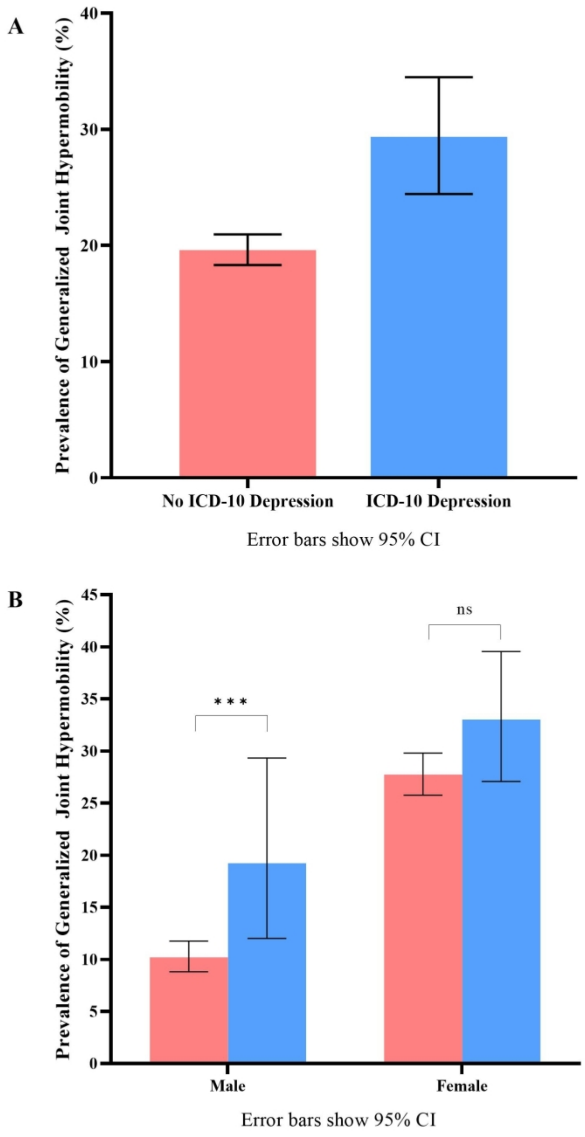

Figure 2: Prevalence of Generalized Joint Hypermobility.
Figure 2A shows the difference in prevalence of GJH in participants who meet diagnostic criteria for ICD-10 depression (blue bar) and those who do not (red bar). Figure 2B shows a significant difference in prevalence of GJH in depressed and non-depressed participants in male but not female participants. Error bars show 95% CI.

137x251mm (300 x 300 DPI)

For peer review only - http://bmjopen.bmj.com/site/about/guidelines.xhtml

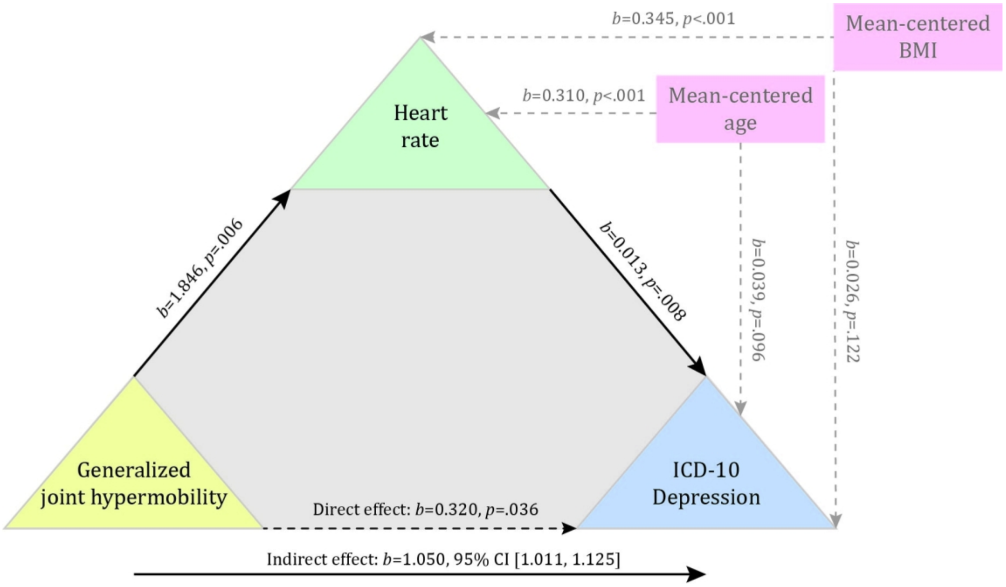

Figure 3: Heart rate mediates the relationship between GJH and depression
This figure displays standardized regression coefficients for the relationship between Generalized Joint Hypermobility and depression as mediated by heart rate (solid black arrows), with the direct (dashed black arrow) and significant indirect effects (solid black arrow). Effects of covariates on heart rate and depression are displayed with grey dashed arrows.

156x115mm (300 x 300 DPI)

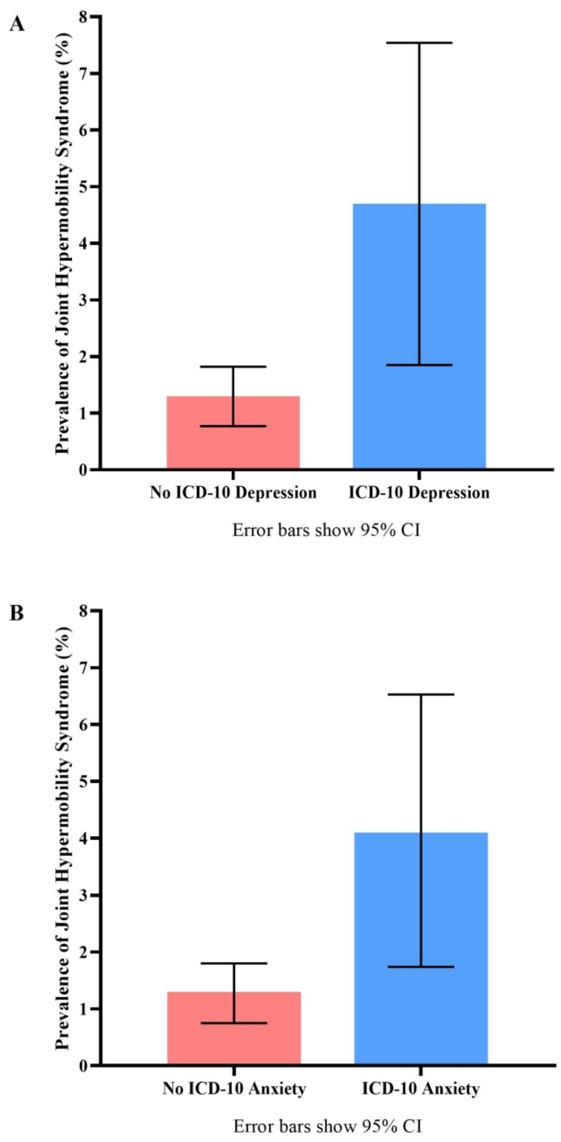

Figure 4: Prevalence of Joint Hypermobility Syndrome
This figure shows the difference in prevalence of JHS in participants who meet ICD-10 criteria (blue bars) for depression (4A), and anxiety (4B), and those who do not meet diagnostic criteria (red bars).

136x259mm (300 x 300 DPI)

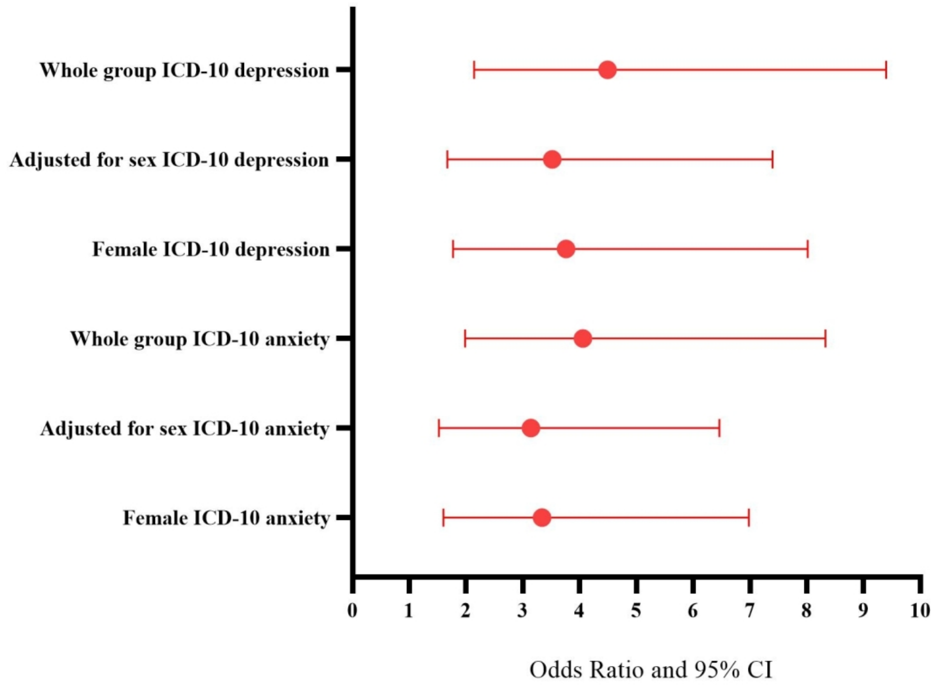

Figure 5: Forest plot of odds ratios
This figure displays the odds rations and their 95% CIs of depression and anxiety given the presence of Joint Hypermobility Syndrome in the whole group, adjusted for sex, and in females only.

140x104mm (300 x 300 DPI)

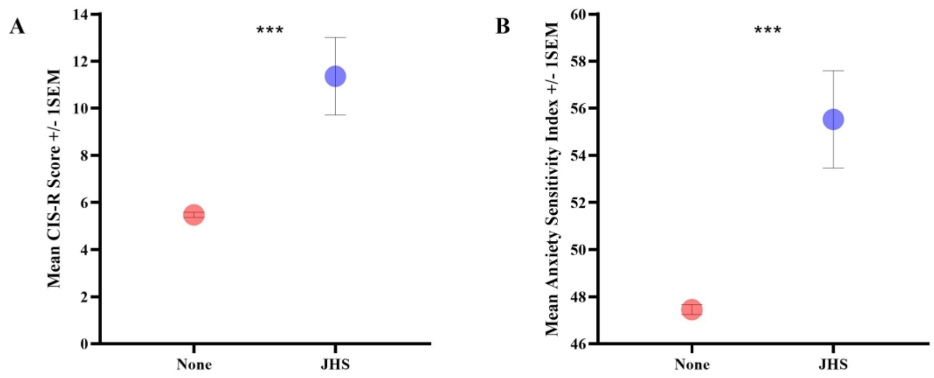

Figure 6: Mean CIS-R scores and Anxiety Sensitivity Index scores
Figure 6A displays the group difference in mean CIS-R (depression) score between participants with (blue dot) and without (red dot) JHS. Figure 6B displays the group difference in mean Anxiety Sensitivity Index score between participants with (blue dot) and without (red dot) JHS. Error bars shows +/- 1 SEM.

214x93mm (300 x 300 DPI)

STROBE Statement—checklist of items that should be included in reports of observational studies

	Item No.	Recommendation	Page No.	Relevant text from manuscript
**Title and abstract**	1	(*a*) Indicate the study's design with a commonly used term in the title or the abstract	1	Variant connective tissue (joint hypermobility) and its relevance to depression and anxiety in adolescents: a cohort-based case-control study
		(*b*) Provide in the abstract an informative and balanced summary of what was done and what was found	2	In a risk analysis, we examined the relationship between generalised joint hypermobility (GJH) at age 14 years with psychiatric symptoms at age 18 years. In an association analysis, we examined the relationship between presence of symptomatic joint hypermobility syndrome (JHS) and ICD-10 diagnoses of depression and anxiety (CIS-R, ASI) at age 18 years. […] Variant collagen, indexed by joint hypermobility, is linked to the emergence of depression and anxiety in adolescence, an effect mediated by autonomic factors in males.
**Introduction**				
Background/rationale	2	Explain the scientific background and rationale for the investigation being reported	3	GJH also is associated with psychiatric conditions and

For peer review only

For peer review only - http://bmjopen.bmj.com/site/about/guidelines.xhtml

				psychological symptoms,[10] where adults with GJH are over-represented among patients expressing common mental disorders; i.e. depression, anxiety and panic.[11] […] Adolescents with PoTS have greater resting heart rates than those without PoTS.[16] […] Nevertheless, in children and adolescents, there remains little information about how joint hypermobility might be associated with common psychiatric conditions.[17]
Objectives	3	State specific objectives, including any prespecified hypotheses	3-4	The objective of this study was to test, for the first time longitudinally, whether there is an association between variant collagen, indexed by joint hypermobility (GJH and JHS) identified in early adolescence, and subsequent risk of depression and anxiety in later adolescence using a population-based birth cohort. We also sought to quantify this relationship and identify potential mediating factors.
**Methods**				
Study design	4	Present key elements of study design early in the paper	4	In this cohort-based case-control study, we used data from the The

For peer review only - http://bmjopen.bmj.com/site/about/guidelines.xhtml

				Avon Longitudinal Study of Parents and Children (ALSPAC) birth cohort to establish risk of psychiatric disorders for children and young people with and without GJH.
Setting	5	Describe the setting, locations, and relevant dates, including periods of recruitment, exposure, follow-up, and data collection	4	The ALSPAC birth cohort follows children of 14062 live births from women living in (the former) Avon County, a geographically defined region in southwest England, with expected dates of delivery between April 1991 and December 1992 (http://www.bristol.ac.uk/alspac).[18] Parents completed regular postal questionnaires about all aspects of their child's health and development from birth. From the age of 7 years, children attended annual assessment clinics, during which they underwent a variety of biomedical and physical tests, face-to-face interviews and standardized assessments of mental health. Data collection follows written informed consent with ethical approval from the ALSPAC Law and Ethics Committee.
Participants	6	(*a*) *Cohort study*—Give the eligibility criteria, and the sources and methods of selection of participants. Describe methods of follow-up	N/A – birth cohort	

For peer review only - http://bmjopen.bmj.com/site/about/guidelines.xhtml

		Case-control study—Give the eligibility criteria, and the sources and methods of case ascertainment and control selection. Give the rationale for the choice of cases and controls<br>*Cross-sectional study*—Give the eligibility criteria, and the sources and methods of selection of participants		
		(*b*) *Cohort study*—For matched studies, give matching criteria and number of exposed and unexposed<br>*Case-control study*—For matched studies, give matching criteria and the number of controls per case		
Variables	7	Clearly define all outcomes, exposures, predictors, potential confounders, and effect modifiers. Give diagnostic criteria, if applicable	4-5	ALSPAC participants underwent measurement of Generalized joint hypermobility (GJH) in a dedicated research clinic, by trained assessors using the modified Beighton 9-point scoring system[6] at two time points; age 14 years and age 18 years. […] Resting heart rate was measured in the same assessment clinic setting, using a POLAR heart rate watch. A 'joint hypermobility syndrome' (JHS) variable was derived to determine if a participant had clinically significant hypermobility at age 18 years. […]Depression and anxiety were measured at age 18 years, using the self-administered computerized version of the Clinical Interview Schedule-Revised (CIS-R). […]

For peer review only - http://bmjopen.bmj.com/site/about/guidelines.xhtml

Data sources/ measurement	8*	For each variable of interest, give sources of data and details of methods of assessment (measurement). Describe comparability of assessment methods if there is more than one group	4-5	Each joint was assessed separately. The fifth metacarpophalangeal joint was scored as hypermobile if it could be extended >90°, the thumb if it could be opposed to the wrist, and the elbows and knees if they could be extended >10°. The spine was scored as hypermobile if both palms could be placed flat on the floor with the knees straight. Scores were recorded for individual joints, and a total score (maximum of 9) was ascertained. In common with other investigators, we used a cut-off point of ≥4 hypermobile joints as a dichotomized definition of the presence of GJH.[9] […]Not all minor criteria were recorded in the ALSPAC cohort at age 18 years, thus JHS was operationalized as meeting two major criteria: the presence of GJH and concomitant chronic widespread pain (CWP)[20]. […]The CIS-R quantifies symptoms to assign International Classification of Diseases-10 (ICD-10) diagnoses of depression and anxiety disorders.[22 23] We derived binary variables for; 1) the presence or absence of a primary diagnosis of major depression,

For peer review only - http://bmjopen.bmj.com/site/about/guidelines.xhtml
For peer review only

				and; 2) presence or absence of a primary diagnosis of any anxiety disorder (including generalized anxiety disorder, panic disorder, agoraphobia, social phobia and specific phobia). In addition, we derived two severity indices; 3) degree of psychiatric symptomatology (i.e., total CIS-R score), and 4) degree of anxiety from the Anxiety Sensitivity Index (total of 'mental and physical concerns' subscale).
Bias	9	Describe any efforts to address potential sources of bias		
Study size	10	Explain how the study size was arrived at	N/A – birth cohort	

Continued on next page

For peer review only

Quantitative variables	11	Explain how quantitative variables were handled in the analyses. If applicable, describe which groupings were chosen and why	5-6	We tested the hypothesis that joint hypermobility was a predictor for subsequent depression or anxiety at age 18 years, by first using logistic regression models to calculate the odds ratios (ORs) and 95% confidence intervals (CI) for depressive disorder and/or anxiety disorder at age 18 years, separately for individuals with and without GJH at age 14 years. Since joint hypermobility is more common in females, analyses for males and females were performed separately. […] We then performed a mediation analysis[25] with presence of depression at age 18 years as the binary outcome variable, GJH at age 14 years as the predictor, and heart rate at age 14 years as a potential mediating variable. […] Analyses were performed separately for males and females
Statistical methods	12	*(a)* Describe all statistical methods, including those used to control for confounding	5-6	We tested the hypothesis that joint hypermobility was a predictor for subsequent

For peer review only

	depression or anxiety at age 18 years, by first using logistic regression models to calculate the odds ratios (ORs) and 95% confidence intervals (CI) for depressive disorder and/or anxiety disorder at age 18 years, separately for individuals with and without GJH at age 14 years. […] To explore possible associations with dysautonomia (PoTS), we conducted separate logistic regression models to test the effect of resting heart rate (as an index of physiological arousal) at age 14 years, on both GJH at aged 14 years and on the later expression of depression and anxiety at age 18 years. […]We then performed a mediation analysis[25] with presence of depression at age 18 years as the binary outcome variable, GJH at age 14 years as the predictor, and heart rate at age 14 years as a potential mediating variable. We used probit regression with full information maximum

For peer review only

likelihood (MPlus estimator command *ML* with Monte Carlo integration). Bootstrapping (n=2000) was applied, with the generation of 95% bias-corrected bootstrap confidence intervals to test inferentially for direct and indirect mediation effects. Analyses were performed separately for males and females. Mediation analyses were controlled for mean-centered age at assessment and body mass index, as both were associated with the potential mediator (heart rate). […]In order to test for an association between symptomatic hypermobility (JHS) and concurrent psychiatric disorders, separate logistic regression models were used to calculate ORs and 95% CI for depression and anxiety disorder at age 18 years in adolescents with and without JHS. Linear regression was used to test for differences between individuals with and without

For peer review only

				JHS in total CIS-R score and anxiety sensitivity.
		(*b*) Describe any methods used to examine subgroups and interactions	5-6	Analyses were performed separately for males and females.
		(*c*) Explain how missing data were addressed	6	For our principle longitudinal analyses, we applied full information maximum likelihood estimation (FIML),[26] where a likelihood function calculates the relationship between a probability estimate based on observed data from variables in the respective analytical model and different estimate values. FIML chooses parameter estimates that maximize this likelihood function based on complete cases, thereby providing robust parameter estimates, despite missing data.[27]
		(*d*) *Cohort study*—If applicable, explain how loss to follow-up was addressed<br>*Case-control study*—If applicable, explain how matching of cases and controls was addressed<br>*Cross-sectional study*—If applicable, describe analytical methods taking account of sampling strategy	N/A – birth cohort	
		(*e*) Describe any sensitivity analyses		
**Results**				
Participants	13*	(a) Report numbers of individuals at each stage of study—eg numbers potentially eligible, examined for eligibility, confirmed eligible, included in the study, completing follow-up, and analysed	6	Table 1, Figure 1

For peer review only

		(b) Give reasons for non-participation at each stage		
		(c) Consider use of a flow diagram		Figure 1
Descriptive data	14*	(a) Give characteristics of study participants (eg demographic, clinical, social) and information on exposures and potential confounders	6	Table 1
		(b) Indicate number of participants with missing data for each variable of interest		Figure 1
		(c) *Cohort study*—Summarise follow-up time (eg, average and total amount)		Figure 1
Outcome data	15*	*Cohort study*—Report numbers of outcome events or summary measures over time		Table 2
		Case-control study—Report numbers in each exposure category, or summary measures of exposure		Table 2
		Cross-sectional study—Report numbers of outcome events or summary measures		
Main results	16	(*a*) Give unadjusted estimates and, if applicable, confounder-adjusted estimates and their precision (eg, 95% confidence interval). Make clear which confounders were adjusted for and why they were included	7-9	Figures 2-6
		(*b*) Report category boundaries when continuous variables were categorized		
		(*c*) If relevant, consider translating estimates of relative risk into absolute risk for a meaningful time period		

Continued on next page

For peer review only - http://bmjopen.bmj.com/site/about/guidelines.xhtml

Other analyses	17	Report other analyses done—eg analyses of subgroups and interactions, and sensitivity analyses	8	A mediation analysis was only conducted in males, for whom the relationship between GJH and depression was significant. Both GJH and heart rate (at age 14 years) were significantly associated with depression (at age 18 years). The mediation analysis showed that the relationship between GJH and depression was mediated by heart rate (Fig. 3).
**Discussion**				
Key results	18	Summarise key results with reference to study objectives	9	Our results extend these findings by showing a link between GJH at age 14 years and subsequent depression in adolescence. There was a significant interaction of sex with significant effects in males only. We also show a mediating effect of basal autonomic physiology (indexed by heart rate) on this relationship. However, we found no longitudinal association between GJH alone and anxiety. This observation is striking as such an association is widely

For peer review only

				reported in adults.[10 11 29] However, symptomatic joint hypermobility (JHS; operationalized here as the presence of GJH and chronic widespread pain) predicted both anxiety and depression in female adolescents and across the whole group when adjusted for sex. Unfortunately, the small number of male cases of JHS did not permit further inference.
Limitations	19	Discuss limitations of the study, taking into account sources of potential bias or imprecision. Discuss both direction and magnitude of any potential bias	9-10	However, it should be noted that the size of effect linking heart rate with hypermobility and depression was small. […] However, in females, additional features of symptomatic hypermobility (JHS), notably chronic widespread pain, are required. […] A more fine-grained assessment of mental health symptoms might have uncovered a broader range of outcomes.
Interpretation	20	Give a cautious overall interpretation of results considering objectives, limitations, multiplicity of analyses, results from similar studies, and other relevant evidence	9-10	Our present finding of a mediating role of heart rate on the association between hypermobility and depression

For peer review only - http://bmjopen.bmj.com/site/about/guidelines.xhtml

				may thus indicate the presence of dysautonomia and constitutional differences in physiological reactivity. […] Our data suggest that depression can be predicted, in males, by the presence of GJH. […] Our study provides evidence for both a longitudinal association between a common variant of connective tissue, hypermobility, and a common psychiatric illness, depression, at age 18 years. We also observed the more direct association between symptomatic joint hypermobility (JHS) and depression and anxiety at age 18 years. […]
Generalisability	21	Discuss the generalisability (external validity) of the study results	10	Together, our findings highlight the importance of a broad biopsychosocial approach to these complex conditions, highlighting the interplay between physical and mental health, with the possibility of screening for hypermobility in adolescence, the judicious use of which may provide opportunities for

For peer review only - http://bmjopen.bmj.com/site/about/guidelines.xhtml
For peer review only

				early intervention to identify and alleviate common psychiatric symptoms.
**Other information**				
Funding	22	Give the source of funding and the role of the funders for the present study and, if applicable, for the original study on which the present article is based	11	Funding for this project came via a fellowship to JAE (MRC MR/K002643/1).

*Give information separately for cases and controls in case-control studies and, if applicable, for exposed and unexposed groups in cohort and cross-sectional studies.

Note: An Explanation and Elaboration article discusses each checklist item and gives methodological background and published examples of transparent reporting. The STROBE checklist is best used in conjunction with this article (freely available on the Web sites of PLoS Medicine at http://www.plosmedicine.org/, Annals of Internal Medicine at http://www.annals.org/, and Epidemiology at http://www.epidem.com/). Information on the STROBE Initiative is available at www.strobe-statement.org.

For peer review only

BMJ Open

Variant connective tissue (joint hypermobility) and its relevance to depression and anxiety in adolescents: a cohort-based case-control study

Journal:	*BMJ Open*
Manuscript ID	bmjopen-2022-066130.R1
Article Type:	Original research
Date Submitted by the Author:	07-Oct-2022
Complete List of Authors:	Eccles, Jessica; Brighton and Sussex Medical School, Department of Neuroscience; Sussex Partnership NHS Foundation Trust<br>Quadt, Lisa; Brighton and Sussex Medical School, Department of Neuroscience; Sussex Partnership NHS Foundation Trust<br>McCarthy, Hannah ; Brighton and Sussex Medical School, Department of Neuroscience; University Hospitals Dorset NHS Foundation Trust<br>Davies, Kevin; Brighton and Sussex Medical School<br>Bond, Rod ; University of Sussex, School of Psychology<br>David, Anthony; UCL, Institute of Mental Health<br>Harrison, Neil; Cardiff University, Brain Research Imaging Centre; Brighton and Sussex Medical School, Department of Neuroscience<br>Critchley, Hugo; Brighton and Sussex Medical School, Neuroscience; Sussex Partnership NHS Foundation Trust
**Primary Subject Heading**:	Mental health
Secondary Subject Heading:	Paediatrics, Rheumatology
Keywords:	MENTAL HEALTH, Paediatric rheumatology < PAEDIATRICS, PAIN MANAGEMENT, Anxiety disorders < PSYCHIATRY, Child & adolescent psychiatry < PSYCHIATRY, Depression & mood disorders < PSYCHIATRY

SCHOLARONE™
Manuscripts

I, the Submitting Author has the right to grant and does grant on behalf of all authors of the Work (as defined in the below author licence), an exclusive licence and/or a non-exclusive licence for contributions from authors who are: i) UK Crown employees; ii) where BMJ has agreed a CC-BY licence shall apply, and/or iii) in accordance with the terms applicable for US Federal Government officers or employees acting as part of their official duties; on a worldwide, perpetual, irrevocable, royalty-free basis to BMJ Publishing Group Ltd ("BMJ") its licensees and where the relevant Journal is co-owned by BMJ to the co-owners of the Journal, to publish the Work in this journal and any other BMJ products and to exploit all rights, as set out in our licence.

The Submitting Author accepts and understands that any supply made under these terms is made by BMJ to the Submitting Author unless you are acting as an employee on behalf of your employer or a postgraduate student of an affiliated institution which is paying any applicable article publishing charge ("APC") for Open Access articles. Where the Submitting Author wishes to make the Work available on an Open Access basis (and intends to pay the relevant APC), the terms of reuse of such Open Access shall be governed by a Creative Commons licence – details of these licences and which Creative Commons licence will apply to this Work are set out in our licence referred to above.

Other than as permitted in any relevant BMJ Author's Self Archiving Policies, I confirm this Work has not been accepted for publication elsewhere, is not being considered for publication elsewhere and does not duplicate material already published. I confirm all authors consent to publication of this Work and authorise the granting of this licence.

For peer review only

Variant connective tissue (joint hypermobility) and its relevance to depression and anxiety in adolescents: a cohort-based case-control study

Jessica A Eccles0000-0002-0062-1216, Lisa Quadt, Hannah E McCarthy, Kevin A Davies, Rod Bond, Anthony S David, Neil A Harrison, Hugo D Critchley

Department of Neuroscience, Brighton and Sussex Medical School, Trafford Centre, BN1 9RY, Brighton, UK
Jessica A Eccles, Clinical Senior Lecturer
Lisa Quadt, Research Fellow
Hannah E McCarthy, Specialty Registrar
Neil A Harrison, Honorary Consultant Professor of Neuropsychiatry
Hugo D Critchley, Head of Department

Sussex Partnership NHS Foundation Trust, Arundel Road, Worthing, BN1 2EP, UK
Jessica A Eccles
Lisa Quadt, Research Fellow
Hugo D Critchley

Royal Bournemouth and Christchurch Hospitals NHS Foundation Trust, Castle Lane East, Dorset, BH7 7DW, UK
Hannah E McCarthy, Specialty Registrar

University Hospitals Sussex NHS Trust, Lyndhurst Road, Worthing, BN11 2DH UK
Jessica A Eccles
Kevin A Davies, Emeritus Foundation Professor of Medicine

Department of Clinical and Experimental Medicine, Brighton and Sussex Medical School, BSMS Teaching Building, Brighton, BN1 9XP, UK
Kevin A Davies, Emeritus Foundation Professor of Medicine

School of Psychology, University of Sussex, Sussex House, Brighton, BN1 9RH, UK
Rod Bond, Emeritus Reader

UCL Institute for Mental Health, University College London, Gower Street, London, WC1E 6BT, UK
Anthony S David, Professor of Mental Health

Cardiff University Brain Research Imaging Centre, Cardiff, CF24 4HQ, UK
Neil A Harrison, Clinical Professor in Neuroimaging

*Correspondence to: Jessica A Eccles, Department of Neuroscience, Brighton and Sussex Medical School, Trafford Centre, University of Sussex, BRIGHTON, BN1 9RY, J.Eccles@bsms.ac.uk

For peer review only - http://bmjopen.bmj.com/site/about/guidelines.xhtml

Abstract

Objective: To test whether variant connective tissue structure, as indicated by the presence of joint hypermobility, poses a developmental risk for mood disorders in adolescence.

Design: Cohort-based case-control study.

Setting: Data from the Avon Longitudinal Study of Parents and Children (ALSPAC) were interrogated.

Participants: 6105 children of the ALSPAC cohort at age 14 years old, of whom 3803 also were assessed when aged 18 years.

Main outcome measures: In a risk analysis, we examined the relationship between generalised joint hypermobility (GJH) at age 14 years with psychiatric symptoms at age 18 years. In an association analysis, we examined the relationship between presence of symptomatic joint hypermobility syndrome (JHS) and International Classification of Diseases (ICD-10) indication of depression and anxiety (Clinical Interview Schedule Revised [CIS-R], Anxiety Sensitivity Index [ASI]) at age 18 years.

Results: GJH was more common in females (n=856, 28%) compared to males (n=319, 11%; OR 3.20 (95% CI 2.78 to 3.68); P<0.001). In males only, GJH at age 14 years was associated with depression at 18 years (OR 2.10 (95%CI 1.17 to 3.76); P=0.013). An index of basal physiological arousal, elevated resting heart rate, mediated this effect. Across genders, the diagnosis of JHS at age 18 years was associated with the presence of depressive disorder (adjusted OR 3.53 (95%CI 1.67 to 7.40); P=0.001), anxiety disorder (adjusted OR 3.14 (95% CI 1.52 to 6.46); P=0.002), level of anxiety (B=8.08, $t(3278)=3.95$; P<0.001), and degree of psychiatric symptomatology (B=5.89, $t(3441)=0.093$; p<0.001).

Conclusions: Variant collagen, indexed by joint hypermobility, is linked to the emergence of depression and anxiety in adolescence, an effect mediated by autonomic factors in males. Recognition of this association may motivate further evaluation, screening, and interventions to mitigate development of psychiatric disorders and improve health outcomes.

Strengths and limitations of this study

- We used data from a large birth-cohort study to investigate whether joint hypermobility increases the risk for depression and anxiety in young people

For peer review only - http://bmjopen.bmj.com/site/about/guidelines.xhtml

- Data points were used from ages 14 and 18, where hypermobility, depression, and anxiety were assessed in the original birth-cohort.
- Available data points were limited due to the design of the original birth-cohort study.

For peer review only

For peer review only - http://bmjopen.bmj.com/site/about/guidelines.xhtml

Introduction

Adolescence is a critical time in psychological and physical development. An individual's trajectory from child into adult is influenced by interacting biological, psychological, environmental, social and cultural factors that determine the subsequent life journey. One quarter of the world's population is aged between 10 and 24 years, and around 75% of psychiatric disorders emerge during adolescence with most fully expressed before age 25 years. Psychiatric disorders in young people now represent half of the global burden of disease, and frequently carry poor long-term outcomes. [1] Longitudinal studies can provide valuable insight into the development of psychiatric disorders. [2] Furthermore, identification of risk factors can inform preventative and early interventional strategies to promote well-being and reduce future morbidity. [3 4]

Generalized joint hypermobility (GJH) is a characteristic marker of hereditary disorders of connective tissue, compromising a matrix of proteins that includes collagens, elastins, fibrillins, and tenascins. [5] Joint hypermobility is typically assessed using the Beighton scale, [6] and is more common in females, declining with age. GJH itself is not necessarily a medical problem, but clinical phenotypes including hypermobile EDS (hEDS; formerly known as EDS hypermobility type/EDS type-III/Joint hypermobility syndrome; JHS), [7] and hypermobility spectrum disorder[8] (including generalized joint hypermobility; GJH) are associated with clinically significant issues. General population estimates show that approximately 20% of adolescents have GJH. [9]

GJH has been robustly associated with psychiatric conditions and psychological symptoms, [10] where adults with GJH are over-represented among patients expressing common mental disorders; i.e. depression, anxiety and panic. [11] A nationwide population-wide cohort study demonstrates association between hypermobility and a variety of psychiatric disorders. [12] Meta-analysis of over 4000 participants suggests odds of demonstrating anxiety in adulthood are 4.39 in hypermobile compared to non-hypermobile people. [13] It is suggested that this relationship in anxiety is related to associated dysautonomia. [14 15]

Correspondingly, neuroimaging studies of individuals with joint hypermobility have identified structural and functional differences within brain regions implicated in emotional arousal, reactivity and feelings, notably amygdala[16] and insula. [17] These observational findings, however, cannot infer a causal relationship, but are of interest in this context.

Differences in cardiovascular autonomic activity (e.g., heart rate) between individuals with and without psychiatric conditions have been observed, where higher resting heart rate

For peer review only - http://bmjopen.bmj.com/site/about/guidelines.xhtml

predicts subsequent psychological symptoms in a large longitudinal cohort study. [18 19] Symptoms of depression are associated with altered autonomic activity, [20 21] such as increases in electrodermal activity, [22] skin temperature and respiratory frequency, [23] and decreased heart rate variability. [24]

Joint hypermobility is also linked to autonomic problems (dysautonomia), [25] often expressed as postural tachycardia syndrome (PoTS), a condition characterized by accelerated heart rate on standing, and other symptoms that overlap phenomenologically with anxiety. [26] Adolescents with PoTS have greater resting heart rates than those without PoTS. [27] In children and adolescents, there remains little information about how joint hypermobility might be associated with common psychiatric conditions. [28] However, a recent cross-sectional case-control study compared 93 children between the ages of 8-15 years with anxiety disorders with 100 age and sex-matched children without anxiety disorders, and found that according to Beighton criteria, 52.7% of children with anxiety disorders had GJH. [29] In contrast, 16% of children in the comparison group had GJH, and age was found to be a risk factor, whereas sex, severity and type of anxiety disorder and ADHD were not predictive of GJH.

The objective of this present study was to test, for the first time longitudinally, whether there is an association between variant collagen, indexed by joint hypermobility (GJH and JHS) identified in early adolescence, and subsequent risk of depression and anxiety in later adolescence using a population-based birth cohort. We also sought to quantify this relationship and identify potential mediating factors.

Methods

Study design and participants

In this cohort-based case-control study, we used data from the The Avon Longitudinal Study of Parents and Children (ALSPAC) birth cohort to establish risk of psychiatric disorders for children and young people with and without GJH. The ALSPAC birth cohort follows children of 14062 live births from women living in (the former) Avon County, a geographically defined region in southwest England, with expected dates of delivery between April 1991 and December 1992 (http://www.bristol.ac.uk/alspac).[30] Parents completed regular postal questionnaires about all aspects of their child's health and development from birth. From the age of 7 years, children attended annual assessment clinics, during which they underwent a variety of biomedical and physical tests, face-to-face interviews and standardized

For peer review only - http://bmjopen.bmj.com/site/about/guidelines.xhtml

assessments of mental health. Data collection followed written informed consent where parents of children under 16 years gave informed consent with the right to withdraw consent at any time. Additional assent from children younger than 16 years was sought at each point of data collection were appropriate. Participants aged 16 years and older gave informed consent (given capacity to make an informed decision. [31]

Analyses for our study were based on two samples, which significantly overlap as they were from the same cohort sample: One sample was used for a predictor model, and the second for an association model (see Fig. 1). The 'risk-set' for the predictor model included 6105 adolescents who underwent a complete joint hypermobility assessment in a clinical setting as 14 years old adolescents. Of this risk set, 5731 adolescents also had heart rate data recorded at age 14. Of these, 3803 adolescents completed assessments for depression and anxiety (Clinical Interview Schedule Revised; CIS-R) [32] aged 18 years. As per ALSPAC data set, these were broad time points and individual age in months varied, and therefore association tests were performed to see if age was a significant variable of interest. The second (association) sample included the 4390 adolescents who completed assessments for both joint hypermobility and the CIS-R aged 18 years. Finally, data from a subset of 3443 adolescents from the association sample, who also completed an assessment for chronic widespread pain (CWP) at the same 18-year time point, were additionally analyzed. We chose the ages of 14 and 18, as these were timepoints in the ALSPAC study at which joint hypermobility was assessed before adulthood. Depression and anxiety were assessed at age 18 together with joint hypermobility, which presented an opportune second time point.

Outcomes

ALSPAC participants underwent measurement of Generalized joint hypermobility (GJH) in a dedicated research clinic, by trained assessors using the modified Beighton 9-point scoring system[6] at two time points; age 14 years and age 18 years. Each joint was assessed separately. The fifth metacarpophalangeal joint was scored as hypermobile if it could be extended >90°, the thumb if it could be opposed to the wrist, and the elbows and knees if they could be extended >10°. The spine was scored as hypermobile if both palms could be placed flat on the floor with the knees straight. Scores were recorded for individual joints, and a total score (maximum of 9) was ascertained. In common with other investigators, we used a cut-off point of ≥4 hypermobile joints as a dichotomized definition of the presence of GJH. [9] Resting heart rate was measured in the same assessment clinic setting, using a POLAR (POLAR Electro, Kempele, Finland) heart rate watch. A 'joint hypermobility syndrome' (JHS) variable was

For peer review only - http://bmjopen.bmj.com/site/about/guidelines.xhtml

derived to determine if a participant had clinically significant hypermobility at age 18 years. JHS diagnosis is typically based on the revised Brighton criteria, [33] and requires a combination of major and minor criteria, comprising GJH in combination with significant musculoskeletal and/or connective tissue symptoms. Not all minor criteria were recorded in the ALSPAC cohort at age 18 years, thus JHS was operationalized as meeting two major criteria: the presence of GJH and concomitant CWP. [34]

Depression and anxiety were measured at age 18 years, using the self-administered computerized version of the Clinical Interview Schedule-Revised (CIS-R). CIS-R is a standardized tool for measuring depression and anxiety in community samples, [32] including this cohort. The CIS-R quantifies symptoms to assign International Classification of Diseases-10 (ICD-10) indications of depression and anxiety disorders, but does not function to exclude other psychiatric conditions which may contribute to or influence symptoms of depression and anxiety. [35 36] We derived binary variables for; 1) the presence or absence of an indication of major depression, and; 2) presence or absence of an indication of any anxiety disorder (including generalized anxiety disorder, panic disorder, agoraphobia, social phobia and specific phobia). In addition, we derived two severity indices; 3) degree of psychiatric symptomatology (i.e., total CIS-R score), and 4) degree of anxiety from the Anxiety Sensitivity Index (ASI; total of 'mental and physical concerns' subscale).

Statistical Analyses

Statistical analyses were conducted in IBM SPSS Statistics Version 26 and MPlus Version 8. [37] We tested the hypothesis that joint hypermobility was a predictor for subsequent depression or anxiety at age 18 years, by first using logistic regression models to calculate the odds ratios (ORs) and 95% confidence intervals (CI) for depressive disorder and/or anxiety disorder at age 18 years, separately for individuals with and without GJH at age 14 years. Since joint hypermobility is more common in females, analyses for males and females were performed separately. To explore possible associations with dysautonomia (PoTS), we conducted separate logistic regression models to test the effect of resting heart rate (as an index of physiological arousal) at age 14 years, on both GJH at aged 14 years and on the later expression of depression and anxiety at age 18 years.

We then performed a mediation analysis[38] with presence of depression at age 18 years as the binary outcome variable, GJH at age 14 years as the predictor, and heart rate at age 14 years as a potential mediating variable. We used probit regression with full information maximum likelihood (MPlus estimator command *ML* with Monte Carlo integration). Bootstrapping

For peer review only - http://bmjopen.bmj.com/site/about/guidelines.xhtml

(n=2000) was applied, with the generation of 95% bias-corrected bootstrap confidence intervals to test inferentially for direct and indirect mediation effects. Analyses were performed separately for males and females. Mediation analyses were controlled for mean-centered age at assessment and body mass index, as both were associated with the potential mediator (heart rate).

In order to test for an association between symptomatic hypermobility (JHS) and concurrent psychiatric disorders, separate logistic regression models were used to calculate ORs and 95% CI for depression and anxiety disorder at age 18 years in adolescents with and without JHS. Linear regression was used to test for differences between individuals with and without JHS in total CIS-R score and anxiety sensitivity. As data were collected at the same time point, these analyses were not subject to maximum likelihood estimation.

For our principle longitudinal analyses, we applied full information maximum likelihood estimation (FIML), [39] where a likelihood function calculates the relationship between a probability estimate based on observed data from variables in the respective analytical model and different estimate values. FIML chooses parameter estimates that maximize this likelihood function based on complete cases, thereby providing robust parameter estimates, despite missing data. [40]

Patient and Public Involvement

This research was motivated by ongoing Patient and Public Involvement (PPI) engagement, in which concerns were regularly expressed by parents of young people with joint hypermobility. PPI members often felt dismissed by medical professionals, and regularly expressed a general need to enhance awareness of joint hypermobility as a risk factor for their children. The underappreciation of its complexity and associated risks were described as barriers to effective care. Our PPI members will help with disseminating our findings among patient groups in an accessible manner.

For peer review only - http://bmjopen.bmj.com/site/about/guidelines.xhtml

Results

Participants

Participant characteristics in the two samples at assessment time points are displayed in Table 1, and participant flow is displayed in Figure 1.

Table 1: Participant Characteristics in the two study samples

**Timepoint**	**Evaluation**	**Participants with available data (n, % male)**	**Age (mean±SD, range)**
**Risk Sample**			
Clinic aged 14	Generalized Joint Hypermobility<br>Heart rate	6105 (49% male)	13·8±0·2 yrs, 12·5-15·2
Clinic aged 18	Depression<br>Anxiety	3803 (45% male)	17·7±0·4 yrs, 16·3-19·3
**Association Sample**			
Clinic aged 18	Joint Hypermobility Syndrome<br>Depression<br>Anxiety	3557 (42% male)	17·7±0·4 yrs, 16·3-19·3

Prevalence of hypermobility and association with age and sex assigned at birth

Of those assessed at 14 years, 1175 (19%) had GJH as reported elsewhere.[9] GJH was more common in females (n=856, 28%) compared to males (n=319, 11%; OR 3.20 [95% CI 2.78-3.68]; *p*<0.001) and not associated with individual variation in age in months.

Of those who had assessments for joint hypermobility and CWP at 18 years, 36 (1%) had both GJH and CWP, and thus met operationalized criteria for JHS. This was significantly more common in females; only 3 were male (OR 8.04 [95% CI 2.46-26.25]; *p*=0.001). JHS was not associated with variation in age.

Risk set analysis

Risk of depression and anxiety at age 18 years according to hypermobility status at age 14 years

Table 2 displays the proportion of participants with GJH at age 14 years in whom depression or anxiety was indexed at age 18 years. Across the whole group, GJH was significantly associated with depression at 18 (OR 1.70 [95% CI 1.31-2.22]; *p*=0.001), where a greater proportion of individuals with depression were hypermobile (had GJH) than those without depression (Table 2, Figure 2A). Additionally, we observed a significant interaction of GJH and sex on depression ($F_{(2,3802)}$=3.73, *p*=0.024), where this effect was significant only in males (OR 2.10 [95% CI 1.17-3.76]; *p*=0.013; Table 2, Figure 2B).

In this analysis, GJH at 14 years was not associated with anxiety at 18 years.

For peer review only - http://bmjopen.bmj.com/site/about/guidelines.xhtml

Table 2: Relationship between hypermobility at age 14 years and depression and anxiety aged 18 years

	Whole group	Male	Female
**No ICD-10 Depression**			
Patients with available data (N)	3510	1627	1883
Proportion of GJH (n, %, 95% CI)	688 (19·6%, 18·31-20·96)	166 (10·2%, 8·82-11·77)	522 (27·74%, 25·76-29·81)
**ICD-10 Depression**			
Patients with available data (N)	293	78	215
Proportion of GJH (n, %, 95% CI)	86 (29·35%, 24·42-34·48)	15 (19·23%, 12·01-29·33)	71 (33·02%, 27·08-39·56)
**No ICD-Anxiety**			
Patients with available data (N)	3437	1604	1832
Proportion of GJH (n, %, 95% CI)	691 (20·10%, 18·79-21·47)	168 (10·47%, 9·06-12·06)	523 (28·55%, 26·53-30·66)
**ICD-10 Anxiety**			
Patients with available data (N)	366	101	265
Proportion of GJH (n, %, 95% CI)	83 (22·68%, 16·69-27·24)	13 (12·87%, 7·68-20·78)	70 (26·41%, 21·48-32·04)

Associations with heart rate at age 14 years

Heart rate was significantly higher in females (M=83.92, SD=11.94) than males (M=78.6, SD=11.44; t(5725)=17.18, p<0.001), and was significantly associated with variations in exact age (months) (r(5729)=-0.32, p=0.016) and Body Mass Index (BMI; r(5722)=0.081, p<0.001) at assessment. After correcting for age and BMI, heart rate at 14 years was significantly associated with both GJH at age 14 years (OR 1.01 [95% CI 1.01-1.02]; p<0.001), and also with depression at 18 years (OR=1.01 [95% CI 1.00-1.02], p=0.007).

Risk of depression at age 18 years

The OR of depression at 18 years in both males and females, given GJH at 14 years, was 1.28 (b=0.246 [95% CI 1.12-1.47, p<0.001). In males, the OR was 1.38 (b=0.032 [95% CI 0.97 to 1.79] p=0.036). There was no significant effect in females only.

Mediation analysis

A mediation analysis was only conducted in males, for whom the relationship between GJH and depression was significant. Both GJH and heart rate (at age 14 years) were significantly associated with depression (at age 18 years). The mediation analysis showed that the relationship between GJH and depression was mediated by heart rate (Fig. 3). This was demonstrated by the bootstrap confidence interval derived from 2000 samples of the indirect effect of heart rate on the relationship between GJH and depression being significant (b=1.05 [95% CI 1.011-1.125]) when controlling for mean centered age and BMI.

For peer review only - http://bmjopen.bmj.com/site/about/guidelines.xhtml

Association set analysis

Symptomatic hypermobility (JHS) and depression at age 18 years

A higher proportion of JHS was found in participants who met ICD-10 criteria for depression (Fig. 4A). Across the whole group, JHS was significantly associated with depression at age 18 years (Fig. 5 shows ORs for this effect, which remained significant when adjusted for sex). Given the low levels of JHS in males (n=3), this analysis was not powered to be undertaken separately but remained significant for females (Fig. 5).

Across the whole group, higher CIS-R scores were present in the JHS group, compared to those without JHS (B=5.89, $t(3442)=5.50$, $p<0.001$; Fig. 6A). In analyses corrected for sex, this association remained (B=4.97, $t(3441)=4.73$, $p<0.001$). In females, higher CIS-R scores were present in those with JHS (M=11.97, SD=10.13), compared to those without JHS (M=6.61, SD=6.88) (B=5.36, $t(1992)=4.40$, $p<0.001$).

Symptomatic hypermobility (JHS) and anxiety at age 18

The prevalence of JHS was greater among participants who met ICD-10 criteria for an anxiety disorder (Fig. 4B). JHS was significantly associated with any anxiety disorder at age 18 years, a relationship that remained significant when adjusted for sex. ORs are displayed in Figure 5.

Across the whole group, an elevated anxiety sensitivity score was observed in individuals with JHS, compared to those without JHS (B=8.08, $t(3278)=3.95$, $p<0.001$; Fig. 6B). This association remained after adjusting for sex (B=6.60, $t(3278)=3.28$, $p=0.001$). In females, higher anxiety sensitivity was present in those with JHS (M=55.94, SD=12.26), compared to those without JHS (M=49.33, SD=11.76; B=-6.61, $t(1907)=-3.10$, $p=0.002$).

Discussion

In adults, the link between variant connective tissue structure (indexed by joint hypermobility) and affective psychopathology is largely established[13] and, interestingly, reinforced by convergent evidence from domestic dogs.[41] Our results extend these findings by showing a link between GJH at age 14 years and subsequent depression in adolescence. There was a significant interaction of sex with significant effects in males only. We also show a mediating effect of basal autonomic physiology (indexed by heart rate) on this relationship. However, we found no longitudinal association between GJH alone and anxiety. This observation is striking as such an association is widely reported in adults.[10 13 42] However, symptomatic joint hypermobility (JHS; operationalized here as the presence of

For peer review only - http://bmjopen.bmj.com/site/about/guidelines.xhtml

GJH and CWP was associated with both anxiety and depression in female adolescents and across the whole group when adjusted for sex.

Variant connective tissue, expressed as joint hypermobility, is characteristically a familial trait. However, no single gene is implicated with consistency. Nevertheless, symptomatic hypermobility (hEDS /HSD/JHS) is considered to be an autosomal dominant trait with incomplete penetrance, variable expressivity, and influenced by sex, since joint hypermobility is more common in females. [43] However, despite this female preponderance, joint hypermobility is also associated with neurodivergence[44] (including Fragile X syndrome, Attention Deficit Hyperactivity Disorder and Autism), which is more commonly diagnosed in males. Importantly, however reported co-occurrences are potentially skewed by underdiagnosis of neurodivergence in females. [45] Neurodivergence also increases the likelihood of clinical anxiety and depression, [46] and it is plausible that this mechanism may account for the observed association between GJH at 14 years and high rates of depression at age 18 years in males.

Hypermobility is associated with dysautonomia, [47] typically postural tachycardia syndrome (PoTS), symptoms of which have phenomenological overlap with anxiety. [26] Adolescents with PoTS have greater resting heart rates than peers without PoTS. [27] Our present finding of a mediating role of heart rate on the association between hypermobility and depression may thus indicate the presence of dysautonomia and constitutional differences in physiological reactivity. Interestingly, a Swedish cohort study also demonstrated that adolescent males with higher basal heart rate are more likely to develop subsequent psychopathology, notably anxiety disorders. [19] However, it should be noted that the size of effect linking heart rate with hypermobility and depression was small.

Our data suggest that depression is associated, in males, with the presence of GJH alone. However, in females, additional features of symptomatic hypermobility (JHS), notably CWP, are required for this association to be significant. One possible explanation for this pattern of results is that the higher rates of anxiety and depression in females reflect susceptibility and perhaps exposure to a wider range of risk factors, which obscures an association with GJH apparent in males.

This study was limited by the measurement instruments used in the ALSPAC cohort study. The categorization and classification of joint hypermobility and symptomatic joint hypermobility remains the subject of substantial debate. [8] Although the Beighton Scale remains the most widely-used approach for measuring hypermobility, there remain considerable questions about its utility, particularly in children and adolescents, [48] and cut-

For peer review only - http://bmjopen.bmj.com/site/about/guidelines.xhtml

offs vary widely. While the CIS-R is a well-established tool to find robust indications of depression and anxiety, it does not control or test for other psychiatric conditions that may influence depressive and anxiety symptomatology. It therefore only an approximation for an indication of the presence of depression and anxiety, albeit a tried and tested one. The ALSPAC data set includes variables for neurodevelopmental conditions like Autism and ADHD, however, these are based on parent reports at age 9 years of the child and were outside of the scope of this work.

Our study provides evidence for both a longitudinal association between a common variant of connective tissue, hypermobility, and a common psychiatric illness, depression, at age 18 years. We also observed the more direct association between symptomatic joint hypermobility (JHS) and depression and anxiety at age 18 years. A more fine-grained assessment of mental health symptoms might have uncovered a broader range of outcomes. Recent years have seen increasing attention in the lay media to joint hypermobility as a single biomedical cause of sometimes profound disability. However, the contribution of underlying variant connective tissue structure is likely to be complex and involves interacting autonomic, psychological and physiological factors. Further systematic scientific inquiry is required. Together, our findings highlight the importance of a broad biopsychosocial approach to these complex conditions, highlighting the interplay between physical and mental health, with the possibility of screening for hypermobility in adolescence, the judicious use of which may provide opportunities for early intervention to identify and alleviate common psychiatric symptoms.

For peer review only

Acknowledgments

We are extremely grateful to all the families who took part in this study, the midwives for their help in recruiting them, and the whole ALSPAC team, which includes interviewers, computer and laboratory technicians, clerical workers, research scientists, volunteers, managers, receptionists and nurses.

Funding

Funding for this project came via a fellowship to JAE (MRC MR/K002643/1).

Author contributions

JE acquired funding, conceptualized and designed the study with support from HC and KD, analysed and managed the data, and drafted the initial manuscript. LQ visualized data and revised the manuscript. RB, HM and LQ supported data analysis. JE, LQ, HM, AD, RB, NH, KD critically reviewed the manuscript, contributed to drafts and approved the final version. We attest all authors meet ICMJE requirements for authorship.

Declaration of Interests

The authors declare no interests.

Data Sharing

All data is available on the ALSPAC data dictionary and variable search tool. (http://www.bristol.ac.uk/alspac/researchers/our-data/).

Ethical Approval

Approval for the original ALSPAC study was obtained from the ALSPAC Ethics and Law Committee (IRB00003312) and Local Research Ethics Committees.

For peer review only - http://bmjopen.bmj.com/site/about/guidelines.xhtml

References

1. Fusar-Poli P. Integrated Mental Health Services for the Developmental Period (0 to 25 Years): A Critical Review of the Evidence. *Front Psychiatry* 2019;10:355. doi: 10.3389/fpsyt.2019.00355 [published Online First: 2019/06/25]
2. Osimo EF, Stochl J, Zammit S, et al. Longitudinal population subgroups of CRP and risk of depression in the ALSPAC birth cohort. *Comprehensive psychiatry* 2020;96:152143. doi: https://doi.org/10.1016/j.comppsych.2019.152143
3. McGorry PD. Is early intervention in the major psychiatric disorders justified? Yes. *BMJ* 2008;337 doi: 10.1136/bmj.a695
4. Colizzi M, Lasalvia A, Ruggeri M. Prevention and early intervention in youth mental health: is it time for a multidisciplinary and trans-diagnostic model for care? *Int J Ment Health Syst* 2020;14:23. doi: 10.1186/s13033-020-00356-9 [published Online First: 2020/04/01]
5. Baeza-Velasco C, Pailhez G, Bulbena A, et al. Joint hypermobility and the heritable disorders of connective tissue: clinical and empirical evidence of links with psychiatry. *General Hospital Psychiatry* 2015;37(1):24-30. doi: 10.1016/j.genhosppsych.2014.10.002
6. Beighton P, Solomon L, Soskolne CL. Articular mobility in an African population. *Annals of the rheumatic diseases* 1973;32(5):413-8.
7. Tinkle BT, Bird HA, Grahame R, et al. The lack of clinical distinction between the hypermobility type of Ehlers-Danlos syndrome and the joint hypermobility syndrome (a.k.a. hypermobility syndrome). *Am J Med Genet A* 2009;149A(11):2368-70. doi: 10.1002/ajmg.a.33070 [published Online First: 2009/10/21]
8. Castori M, Tinkle B, Levy H, et al. A framework for the classification of joint hypermobility and related conditions. *American journal of medical genetics* 2017;175(1):148-57. doi: 10.1002/ajmg.c.31539
9. Clinch J, Deere K, Sayers A, et al. Epidemiology of generalized joint laxity (hypermobility) in fourteen-year-old children from the UK: A population-based evaluation. *Arthritis & Rheumatism* 2011;63(9):2819-27. doi: 10.1002/art.30435
10. Sharp HEC, Critchley HD, Eccles JA. Connecting brain and body: Transdiagnostic relevance of connective tissue variants to neuropsychiatric symptom expression. *World J Psychiatry* 2021;11(10):805-20. doi: 10.5498/wjp.v11.i10.805 [published Online First: 2021/11/05]
11. Bulbena A, Baeza-Velasco C, Bulbena-Cabre A, et al. Psychiatric and psychological aspects in the Ehlers-Danlos syndromes. *American journal of medical genetics* 2017;175(1):237-45. doi: 10.1002/ajmg.c.31544
12. Cederlöf M, Larsson H, Lichtenstein P, et al. Nationwide population-based cohort study of psychiatric disorders in individuals with Ehlers-Danlos syndrome or hypermobility syndrome and their siblings. *BMC Psychiatry* 2016;16:207. doi: 10.1186/s12888-016-0922-6
13. Smith TO, Easton V, Bacon H, et al. The relationship between benign joint hypermobility syndrome and psychological distress: a systematic review and meta-analysis. *Rheumatology (Oxford)* 2014;53(1):114-22. doi: 10.1093/rheumatology/ket317
ket317 [pii] [published Online First: 2013/10/02]
14. Csecs JLL, Dowell NG, Savage GK, et al. Variant connective tissue (joint hypermobility) and dysautonomia are associated with multimorbidity at the intersection between physical and psychological health. *American Journal of Medical Genetics Part C: Seminars in Medical Genetics* 2021;187(4):500-09. doi: 10.1002/ajmg.c.31957

For peer review only - http://bmjopen.bmj.com/site/about/guidelines.xhtml

15. Eccles JA, Owens AP, Mathias CJ, et al. Neurovisceral phenotypes in the expression of psychiatric symptoms. *Frontiers in Neuroscience* 2015;9(4) doi: 10.3389/fnins.2015.00004
16. Eccles JA, Beacher FD, Gray MA, et al. Brain structure and joint hypermobility: relevance to the expression of psychiatric symptoms. *British Journal of Psychiatry* 2012;200(6):508-09. doi: 10.1192/bjp.bp.111.092460
17. Mallorqui-Bague N, Garfinkel SN, Engels M, et al. Neuroimaging and psychophysiological investigation of the link between anxiety, enhanced affective reactivity and interoception in people with joint hypermobility. *Front Psychol* 2014;5:1162. doi: 10.3389/fpsyg.2014.01162 [published Online First: 2014/10/30]
18. Kendler KS, Lönn SL, Sundquist J, et al. The causal nature of the association between resting pulse in late adolescence and risk for internalizing and externalizing disorders: a co-relative analysis in a national male Swedish sample. *Psychol Med* 2021;51(11):1822-28. doi: 10.1017/s0033291720000549 [published Online First: 20200324]
19. Latvala A, Kuja-Halkola R, Ruck C, et al. Association of Resting Heart Rate and Blood Pressure in Late Adolescence With Subsequent Mental Disorders: A Longitudinal Population Study of More Than 1 Million Men in Sweden. *JAMA Psychiatry* 2016;73(12):1268-75. doi: 10.1001/jamapsychiatry.2016.2717
20. Critchley HD, Nagai Y. How Emotions Are Shaped by Bodily States. *Emotion Review* 2012;4(2):163-68. doi: 10.1177/1754073911430132
21. Ranfaing S, De Zorzi L, Honoré J, et al. Attention orientation to pleasantness and depressive symptomatology predict autonomic reactivity. *Cognition and Emotion* 2021;35(6):1203-13. doi: 10.1080/02699931.2021.1929852
22. Branković SB. System identification of skin conductance response in depression - an attempt to probe the neurochemistry of limbic system. *Psychiatr Danub* 2008;20(3):310-22.
23. Wenzler S, Hagen M, Tarvainen MP, et al. Intensified emotion perception in depression: Differences in physiological arousal and subjective perceptions. *Psychiatry Research* 2017;253:303-10. doi: https://doi.org/10.1016/j.psychres.2017.03.040
24. Kemp AH, Quintana DS, Gray MA, et al. Impact of Depression and Antidepressant Treatment on Heart Rate Variability: A Review and Meta-Analysis. *Biological Psychiatry* 2010;67(11):1067-74. doi: https://doi.org/10.1016/j.biopsych.2009.12.012
25. Celletti C, Camerota F, Castori M, et al. Orthostatic Intolerance and Postural Orthostatic Tachycardia Syndrome in Joint Hypermobility Syndrome/Ehlers-Danlos Syndrome, Hypermobility Type: Neurovegetative Dysregulation or Autonomic Failure? *BioMed research international* 2017;2017:9161865. doi: 10.1155/2017/9161865 [published Online First: 2017/03/14]
26. Mathias CJ, Low DA, Iodice V, et al. Postural tachycardia syndrome--current experience and concepts. *Nat Rev Neurol* 2012;8(1):22-34. doi: 10.1038/nrneurol.2011.187
nrneurol.2011.187 [pii] [published Online First: 2011/12/07]
27. Singer W, Sletten DM, Opfer-Gehrking TL, et al. Postural tachycardia in children and adolescents: what is abnormal? *The Journal of pediatrics* 2012;160(2):222-6. doi: 10.1016/j.jpeds.2011.08.054
28. Ezpeleta L, Navarro JB, Osa N, et al. Joint Hypermobility Classes in 9-Year-Old Children from the General Population and Anxiety Symptoms. *J Dev Behav Pediatr* 2018;39(6):481-88. doi: 10.1097/DBP.0000000000000577 [published Online First: 2018/05/31]

For peer review only - http://bmjopen.bmj.com/site/about/guidelines.xhtml

29. Javadi Parvaneh V, Modaress S, Zahed G, et al. Prevalence of generalized joint hypermobility in children with anxiety disorders. *BMC Musculoskeletal Disorders* 2020;21(1) doi: 10.1186/s12891-020-03377-0
30. Boyd A, Golding J, Macleod J, et al. Cohort Profile: The 'Children of the 90s'—the index offspring of the Avon Longitudinal Study of Parents and Children. *International Journal of Epidemiology* 2013;42(1):111-27. doi: 10.1093/ije/dys064
31. Northstone K, Lewcock M, Groom A, et al. The Avon Longitudinal Study of Parents and Children (ALSPAC): an update on the enrolled sample of index children in 2019. *Wellcome Open Research* 2019;4:51. doi: 10.12688/wellcomeopenres.15132.1
32. Lewis G, Pelosi AJ, Araya R, et al. Measuring psychiatric disorder in the community: a standardized assessment for use by lay interviewers. *Psychol Med* 1992;22(2):465-86. [published Online First: 1992/05/01]
33. Grahame R, Bird HA, Child A. The revised (Brighton 1998) criteria for the diagnosis of benign joint hypermobility syndrome (BJHS). *J Rheumatol* 2000;27(7):1777-9.
34. Deere KC, Clinch J, Holliday K, et al. Obesity is a risk factor for musculoskeletal pain in adolescents: findings from a population-based cohort. *Pain* 2012;153(9):1932-8. doi: 10.1016/j.pain.2012.06.006
S0304-3959(12)00360-0 [pii] [published Online First: 2012/07/19]
35. Lewis G, Araya R. Classification, disability and the public health agenda. *Br Med Bull* 2001;57:3-15. [published Online First: 2001/11/27]
36. Brugha TS, Bebbington PE, Jenkins R, et al. Cross validation of a general population survey diagnostic interview: a comparison of CIS-R with SCAN ICD-10 diagnostic categories. *Psychol Med* 1999;29(5):1029-42. [published Online First: 1999/11/27]
37. Muthén L, Muthén B. Statistical Analysis with Latent Variables: Mplus User's Guide: Muthén & Muthén, Los Angeles, CA, 2012.
38. Muthen B, Asparouhov T. Causal Effects in Mediation Modeling: An Introduction With Applications to Latent Variables. *Struct Equ Modeling* 2015;22(1):12-23. doi: 10.1080/10705511.2014.935843
39. Enders CK. Applied missing data analysis. New York, NY, US: Guilford Press 2010.
40. Newman DA. Missing Data: Five Practical Guidelines. *Organizational Research Methods* 2014;17(4):372-411. doi: 10.1177/1094428114548590
41. Bowen J, Fatjo J, Serpell JA, et al. First evidence for an association between joint hypermobility and excitability in a non-human species, the domestic dog. *Sci Rep* 2019;9(1):8629. doi: 10.1038/s41598-019-45096-0 [published Online First: 2019/06/15]
42. Bulbena A, Gago J, Pailhez G, et al. Joint hypermobility syndrome is a risk factor trait for anxiety disorders: a 15-year follow-up cohort study. *Gen Hosp Psychiatry* 2011;33(4):363-70. doi: 10.1016/j.genhosppsych.2011.03.004
43. Castori M. Ehlers-danlos syndrome, hypermobility type: an underdiagnosed hereditary connective tissue disorder with mucocutaneous, articular, and systemic manifestations. *ISRN Dermatol* 2012;2012:751768. doi: 10.5402/2012/751768
44. Csecs JLL, Iodice V, Rae CL, et al. Joint Hypermobility Links Neurodivergence to Dysautonomia and Pain. *Frontiers in Psychiatry* 2022;12 doi: 10.3389/fpsyt.2021.786916
45. Allely CS. Understanding and recognising the female phenotype of autism spectrum disorder and the "camouflage" hypothesis: a systematic PRISMA review. *Advances in Autism* 2019;5(1):14-37. doi: 10.1108/aia-09-2018-0036
46. Katzman MA, Bilkey TS, Chokka PR, et al. Adult ADHD and comorbid disorders: clinical implications of a dimensional approach. *BMC Psychiatry* 2017;17(1):302. doi: 10.1186/s12888-017-1463-3

For peer review only - http://bmjopen.bmj.com/site/about/guidelines.xhtml

47. Gazit Y, Nahir AM, Grahame R, et al. Dysautonomia in the joint hypermobility syndrome. *Am J Med* 2003;115(1):33-40. doi: S0002934303002353 [pii] [published Online First: 2003/07/18]
48. Simmonds J. Generalized joint hypermobility: a timely population study and proposal for Beighton cut-offs: Beighton cut-offs for generalized joint hypermobility. *Rheumatology (Oxford)* 2017 doi: 10.1093/rheumatology/kex190

For peer review only

For peer review only - http://bmjopen.bmj.com/site/about/guidelines.xhtml

Figure Legends

Figure 1: Flow diagram of participants in study samples

Figure 2: Prevalence of Generalized Joint Hypermobility.

Figure 2A shows the difference in prevalence of GJH in participants who meet diagnostic criteria for ICD-10 depression (blue bar) and those who do not (red bar). Figure 2B shows a significant difference in prevalence of GJH in depressed and non-depressed participants in male but not female participants. Error bars show 95% CI.

Figure 3: Heart rate mediates the relationship between GJH and depression

This figure displays standardized regression coefficients for the relationship between Generalized Joint Hypermobility and depression as mediated by heart rate (solid black arrows), with the direct (dashed black arrow) and significant indirect effects (solid black arrow). Effects of covariates on heart rate and depression are displayed with grey dashed arrows.

Figure 4: Prevalence of Joint Hypermobility Syndrome

This figure shows the difference in prevalence of JHS in participants who meet ICD-10 criteria (blue bars) for depression (4A), and anxiety (4B), and those who do not meet diagnostic criteria (red bars).

Figure 5: Forest plot of odds ratios

This figure displays the odds rations and their 95% CIs of depression and anxiety given the presence of Joint Hypermobility Syndrome in the whole group, adjusted for sex, and in females only.

Figure 6: Mean CIS-R scores and Anxiety Sensitivity Index scores

Figure 6A displays the group difference in mean CIS-R (depression) score between participants with (blue dot) and without (red dot) JHS. Figure 6B displays the group difference in mean Anxiety Sensitivity Index score between participants with (blue dot) and without (red dot) JHS. Error bars shows +/- 1 SEM.

For peer review only

For peer review only - http://bmjopen.bmj.com/site/about/guidelines.xhtml

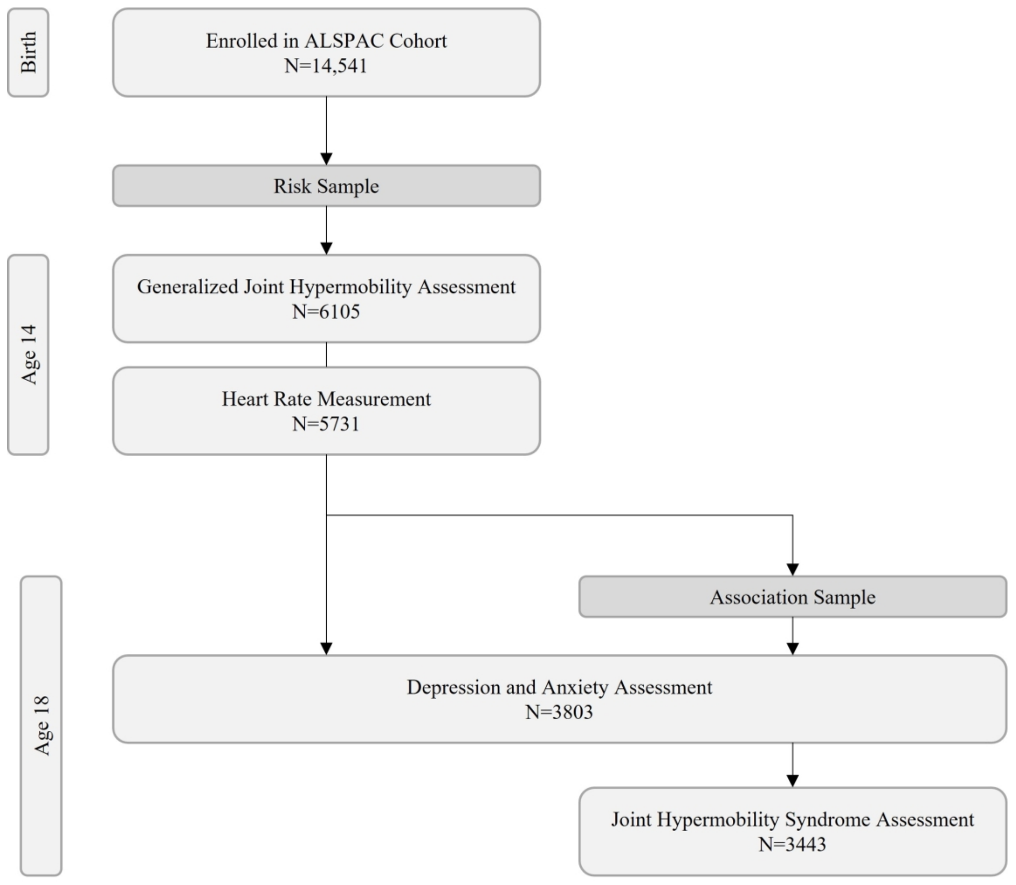

Figure 1: Flow diagram of participants in study samples

169x162mm (330 x 330 DPI)

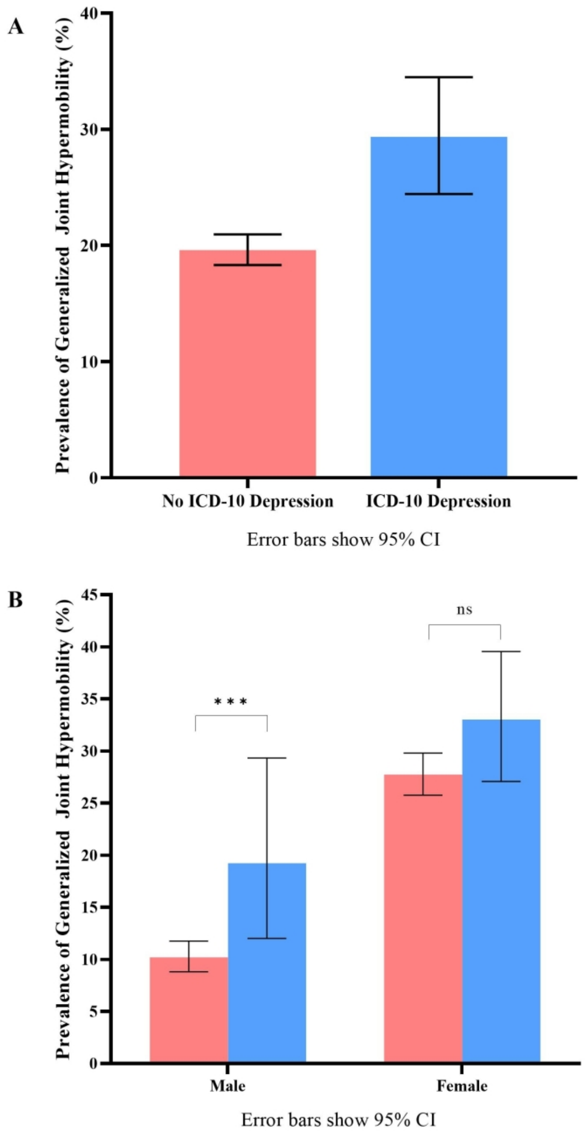

Figure 2: Prevalence of Generalized Joint Hypermobility.
Figure 2A shows the difference in prevalence of GJH in participants who meet diagnostic criteria for ICD-10 depression (blue bar) and those who do not (red bar). Figure 2B shows a significant difference in prevalence of GJH in depressed and non-depressed participants in male but not female participants. Error bars show 95% CI.

137x251mm (300 x 300 DPI)

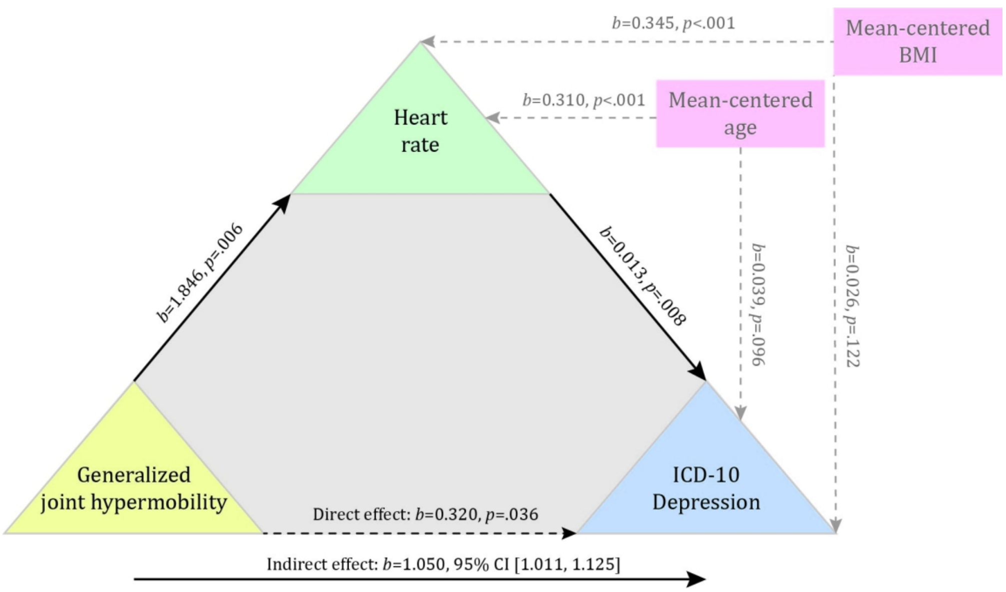

Figure 3: Heart rate mediates the relationship between GJH and depression
This figure displays standardized regression coefficients for the relationship between Generalized Joint Hypermobility and depression as mediated by heart rate (solid black arrows), with the direct (dashed black arrow) and significant indirect effects (solid black arrow). Effects of covariates on heart rate and depression are displayed with grey dashed arrows.

156x115mm (300 x 300 DPI)

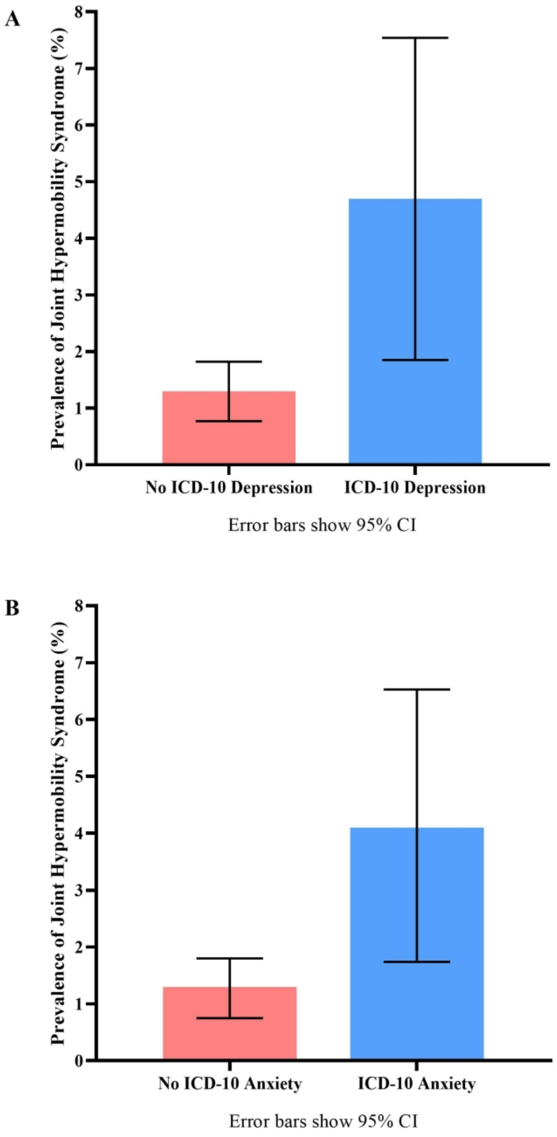

Figure 4: Prevalence of Joint Hypermobility Syndrome
This figure shows the difference in prevalence of JHS in participants who meet ICD-10 criteria (blue bars) for depression (4A), and anxiety (4B), and those who do not meet diagnostic criteria (red bars).

136x259mm (300 x 300 DPI)

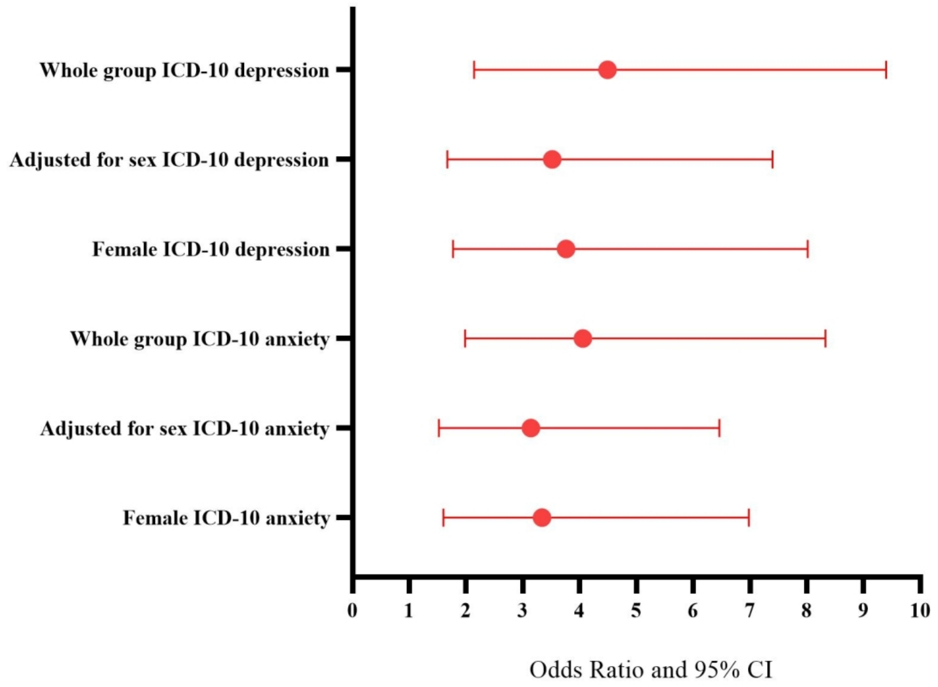

Figure 5: Forest plot of odds ratios
This figure displays the odds rations and their 95% CIs of depression and anxiety given the presence of Joint Hypermobility Syndrome in the whole group, adjusted for sex, and in females only.

140x104mm (300 x 300 DPI)

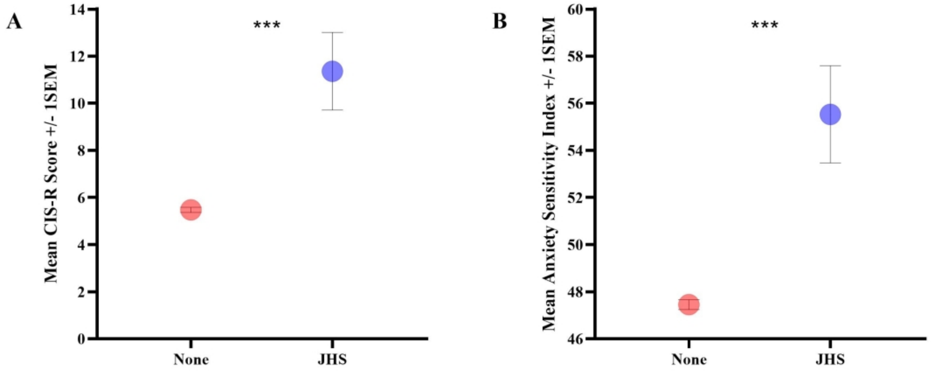

Figure 6: Mean CIS-R scores and Anxiety Sensitivity Index scores
Figure 6A displays the group difference in mean CIS-R (depression) score between participants with (blue dot) and without (red dot) JHS. Figure 6B displays the group difference in mean Anxiety Sensitivity Index score between participants with (blue dot) and without (red dot) JHS. Error bars shows +/- 1 SEM.

214x93mm (300 x 300 DPI)

STROBE Statement—checklist of items that should be included in reports of observational studies

	Item No.	Recommendation	Page No.	Relevant text from manuscript
**Title and abstract**	1	(*a*) Indicate the study's design with a commonly used term in the title or the abstract	1	Variant connective tissue (joint hypermobility) and its relevance to depression and anxiety in adolescents: a cohort-based case-control study
		(*b*) Provide in the abstract an informative and balanced summary of what was done and what was found	2	In a risk analysis, we examined the relationship between generalised joint hypermobility (GJH) at age 14 years with psychiatric symptoms at age 18 years. In an association analysis, we examined the relationship between presence of symptomatic joint hypermobility syndrome (JHS) and ICD-10 diagnoses of depression and anxiety (CIS-R, ASI) at age 18 years. […] Variant collagen, indexed by joint hypermobility, is linked to the emergence of depression and anxiety in adolescence, an effect mediated by autonomic factors in males.
**Introduction**				
Background/rationale	2	Explain the scientific background and rationale for the investigation being reported	3	GJH also is associated with psychiatric conditions and

For peer review only

				psychological symptoms,[10] where adults with GJH are over-represented among patients expressing common mental disorders; i.e. depression, anxiety and panic.[11] […] Adolescents with PoTS have greater resting heart rates than those without PoTS.[16] […] Nevertheless, in children and adolescents, there remains little information about how joint hypermobility might be associated with common psychiatric conditions.[17]
Objectives	3	State specific objectives, including any prespecified hypotheses	3-4	The objective of this study was to test, for the first time longitudinally, whether there is an association between variant collagen, indexed by joint hypermobility (GJH and JHS) identified in early adolescence, and subsequent risk of depression and anxiety in later adolescence using a population-based birth cohort. We also sought to quantify this relationship and identify potential mediating factors.
**Methods**				
Study design	4	Present key elements of study design early in the paper	4	In this cohort-based case-control study, we used data from the The

For peer review only - http://bmjopen.bmj.com/site/about/guidelines.xhtml

				Avon Longitudinal Study of Parents and Children (ALSPAC) birth cohort to establish risk of psychiatric disorders for children and young people with and without GJH.
Setting	5	Describe the setting, locations, and relevant dates, including periods of recruitment, exposure, follow-up, and data collection	4	The ALSPAC birth cohort follows children of 14062 live births from women living in (the former) Avon County, a geographically defined region in southwest England, with expected dates of delivery between April 1991 and December 1992 (http://www.bristol.ac.uk/alspac).[18] Parents completed regular postal questionnaires about all aspects of their child's health and development from birth. From the age of 7 years, children attended annual assessment clinics, during which they underwent a variety of biomedical and physical tests, face-to-face interviews and standardized assessments of mental health. Data collection follows written informed consent with ethical approval from the ALSPAC Law and Ethics Committee.
Participants	6	(*a*) *Cohort study*—Give the eligibility criteria, and the sources and methods of selection of participants. Describe methods of follow-up	N/A – birth cohort	

For peer review only - http://bmjopen.bmj.com/site/about/guidelines.xhtml

For peer review only

		Case-control study—Give the eligibility criteria, and the sources and methods of case ascertainment and control selection. Give the rationale for the choice of cases and controls<br>*Cross-sectional study*—Give the eligibility criteria, and the sources and methods of selection of participants		
		(*b*) *Cohort study*—For matched studies, give matching criteria and number of exposed and unexposed<br>*Case-control study*—For matched studies, give matching criteria and the number of controls per case		
Variables	7	Clearly define all outcomes, exposures, predictors, potential confounders, and effect modifiers. Give diagnostic criteria, if applicable	4-5	ALSPAC participants underwent measurement of Generalized joint hypermobility (GJH) in a dedicated research clinic, by trained assessors using the modified Beighton 9-point scoring system[6] at two time points; age 14 years and age 18 years. […] Resting heart rate was measured in the same assessment clinic setting, using a POLAR heart rate watch. A 'joint hypermobility syndrome' (JHS) variable was derived to determine if a participant had clinically significant hypermobility at age 18 years. […]Depression and anxiety were measured at age 18 years, using the self-administered computerized version of the Clinical Interview Schedule-Revised (CIS-R). […]

For peer review only

Data sources/ measurement	8*	For each variable of interest, give sources of data and details of methods of assessment (measurement). Describe comparability of assessment methods if there is more than one group	4-5	Each joint was assessed separately. The fifth metacarpophalangeal joint was scored as hypermobile if it could be extended >90°, the thumb if it could be opposed to the wrist, and the elbows and knees if they could be extended >10°. The spine was scored as hypermobile if both palms could be placed flat on the floor with the knees straight. Scores were recorded for individual joints, and a total score (maximum of 9) was ascertained. In common with other investigators, we used a cut-off point of ≥4 hypermobile joints as a dichotomized definition of the presence of GJH.[9] […]Not all minor criteria were recorded in the ALSPAC cohort at age 18 years, thus JHS was operationalized as meeting two major criteria: the presence of GJH and concomitant chronic widespread pain (CWP)[20]. […]The CIS-R quantifies symptoms to assign International Classification of Diseases-10 (ICD-10) diagnoses of depression and anxiety disorders.[22 23] We derived binary variables for; 1) the presence or absence of a primary diagnosis of major depression,

For peer review only - http://bmjopen.bmj.com/site/about/guidelines.xhtml
For peer review only

				and; 2) presence or absence of a primary diagnosis of any anxiety disorder (including generalized anxiety disorder, panic disorder, agoraphobia, social phobia and specific phobia). In addition, we derived two severity indices; 3) degree of psychiatric symptomatology (i.e., total CIS-R score), and 4) degree of anxiety from the Anxiety Sensitivity Index (total of 'mental and physical concerns' subscale).
Bias	9	Describe any efforts to address potential sources of bias		
Study size	10	Explain how the study size was arrived at	N/A – birth cohort	

Continued on next page

For peer review only

Quantitative variables	11	Explain how quantitative variables were handled in the analyses. If applicable, describe which groupings were chosen and why	5-6	We tested the hypothesis that joint hypermobility was a predictor for subsequent depression or anxiety at age 18 years, by first using logistic regression models to calculate the odds ratios (ORs) and 95% confidence intervals (CI) for depressive disorder and/or anxiety disorder at age 18 years, separately for individuals with and without GJH at age 14 years. Since joint hypermobility is more common in females, analyses for males and females were performed separately. […] We then performed a mediation analysis[25] with presence of depression at age 18 years as the binary outcome variable, GJH at age 14 years as the predictor, and heart rate at age 14 years as a potential mediating variable. […] Analyses were performed separately for males and females
Statistical methods	12	(*a*) Describe all statistical methods, including those used to control for confounding	5-6	We tested the hypothesis that joint hypermobility was a predictor for subsequent

For peer review only - http://bmjopen.bmj.com/site/about/guidelines.xhtml

	depression or anxiety at age 18 years, by first using logistic regression models to calculate the odds ratios (ORs) and 95% confidence intervals (CI) for depressive disorder and/or anxiety disorder at age 18 years, separately for individuals with and without GJH at age 14 years. […] To explore possible associations with dysautonomia (PoTS), we conducted separate logistic regression models to test the effect of resting heart rate (as an index of physiological arousal) at age 14 years, on both GJH at aged 14 years and on the later expression of depression and anxiety at age 18 years. […]We then performed a mediation analysis[25] with presence of depression at age 18 years as the binary outcome variable, GJH at age 14 years as the predictor, and heart rate at age 14 years as a potential mediating variable. We used probit regression with full information maximum

For peer review only - http://bmjopen.bmj.com/site/about/guidelines.xhtml
For peer review only

likelihood (MPlus estimator command *ML* with Monte Carlo integration). Bootstrapping (n=2000) was applied, with the generation of 95% bias-corrected bootstrap confidence intervals to test inferentially for direct and indirect mediation effects. Analyses were performed separately for males and females. Mediation analyses were controlled for mean-centered age at assessment and body mass index, as both were associated with the potential mediator (heart rate). [...]In order to test for an association between symptomatic hypermobility (JHS) and concurrent psychiatric disorders, separate logistic regression models were used to calculate ORs and 95% CI for depression and anxiety disorder at age 18 years in adolescents with and without JHS. Linear regression was used to test for differences between individuals with and without

For peer review only - http://bmjopen.bmj.com/site/about/guidelines.xhtml
For peer review only

				JHS in total CIS-R score and anxiety sensitivity.
		(*b*) Describe any methods used to examine subgroups and interactions	5-6	Analyses were performed separately for males and females.
		(*c*) Explain how missing data were addressed	6	For our principle longitudinal analyses, we applied full information maximum likelihood estimation (FIML),[26] where a likelihood function calculates the relationship between a probability estimate based on observed data from variables in the respective analytical model and different estimate values. FIML chooses parameter estimates that maximize this likelihood function based on complete cases, thereby providing robust parameter estimates, despite missing data.[27]
		(*d*) *Cohort study*—If applicable, explain how loss to follow-up was addressed<br>*Case-control study*—If applicable, explain how matching of cases and controls was addressed<br>*Cross-sectional study*—If applicable, describe analytical methods taking account of sampling strategy	N/A – birth cohort	
		(*e*) Describe any sensitivity analyses		
**Results**				
Participants	13*	(a) Report numbers of individuals at each stage of study—eg numbers potentially eligible, examined for eligibility, confirmed eligible, included in the study, completing follow-up, and analysed	6	Table 1, Figure 1

For peer review only

		(b) Give reasons for non-participation at each stage		
		(c) Consider use of a flow diagram		Figure 1
Descriptive data	14*	(a) Give characteristics of study participants (eg demographic, clinical, social) and information on exposures and potential confounders	6	Table 1
		(b) Indicate number of participants with missing data for each variable of interest		Figure 1
		(c) *Cohort study*—Summarise follow-up time (eg, average and total amount)		Figure 1
Outcome data	15*	*Cohort study*—Report numbers of outcome events or summary measures over time		Table 2
		Case-control study—Report numbers in each exposure category, or summary measures of exposure		Table 2
		Cross-sectional study—Report numbers of outcome events or summary measures		
Main results	16	(*a*) Give unadjusted estimates and, if applicable, confounder-adjusted estimates and their precision (eg, 95% confidence interval). Make clear which confounders were adjusted for and why they were included	7-9	Figures 2-6
		(*b*) Report category boundaries when continuous variables were categorized		
		(*c*) If relevant, consider translating estimates of relative risk into absolute risk for a meaningful time period		

Continued on next page

For peer review only - http://bmjopen.bmj.com/site/about/guidelines.xhtml

Other analyses	17	Report other analyses done—eg analyses of subgroups and interactions, and sensitivity analyses	8	A mediation analysis was only conducted in males, for whom the relationship between GJH and depression was significant. Both GJH and heart rate (at age 14 years) were significantly associated with depression (at age 18 years). The mediation analysis showed that the relationship between GJH and depression was mediated by heart rate (Fig. 3).
**Discussion**				
Key results	18	Summarise key results with reference to study objectives	9	Our results extend these findings by showing a link between GJH at age 14 years and subsequent depression in adolescence. There was a significant interaction of sex with significant effects in males only. We also show a mediating effect of basal autonomic physiology (indexed by heart rate) on this relationship. However, we found no longitudinal association between GJH alone and anxiety. This observation is striking as such an association is widely

For peer review only

				reported in adults.[10 11 29] However, symptomatic joint hypermobility (JHS; operationalized here as the presence of GJH and chronic widespread pain) predicted both anxiety and depression in female adolescents and across the whole group when adjusted for sex. Unfortunately, the small number of male cases of JHS did not permit further inference.
Limitations	19	Discuss limitations of the study, taking into account sources of potential bias or imprecision. Discuss both direction and magnitude of any potential bias	9-10	However, it should be noted that the size of effect linking heart rate with hypermobility and depression was small. […] However, in females, additional features of symptomatic hypermobility (JHS), notably chronic widespread pain, are required. […] A more fine-grained assessment of mental health symptoms might have uncovered a broader range of outcomes.
Interpretation	20	Give a cautious overall interpretation of results considering objectives, limitations, multiplicity of analyses, results from similar studies, and other relevant evidence	9-10	Our present finding of a mediating role of heart rate on the association between hypermobility and depression

For peer review only - http://bmjopen.bmj.com/site/about/guidelines.xhtml
For peer review only

				may thus indicate the presence of dysautonomia and constitutional differences in physiological reactivity. […] Our data suggest that depression can be predicted, in males, by the presence of GJH. […] Our study provides evidence for both a longitudinal association between a common variant of connective tissue, hypermobility, and a common psychiatric illness, depression, at age 18 years. We also observed the more direct association between symptomatic joint hypermobility (JHS) and depression and anxiety at age 18 years. […]
Generalisability	21	Discuss the generalisability (external validity) of the study results	10	Together, our findings highlight the importance of a broad biopsychosocial approach to these complex conditions, highlighting the interplay between physical and mental health, with the possibility of screening for hypermobility in adolescence, the judicious use of which may provide opportunities for

For peer review only

				early intervention to identify and alleviate common psychiatric symptoms.
**Other information**				
Funding	22	Give the source of funding and the role of the funders for the present study and, if applicable, for the original study on which the present article is based	11	Funding for this project came via a fellowship to JAE (MRC MR/K002643/1).

*Give information separately for cases and controls in case-control studies and, if applicable, for exposed and unexposed groups in cohort and cross-sectional studies.

Note: An Explanation and Elaboration article discusses each checklist item and gives methodological background and published examples of transparent reporting. The STROBE checklist is best used in conjunction with this article (freely available on the Web sites of PLoS Medicine at http://www.plosmedicine.org/, Annals of Internal Medicine at http://www.annals.org/, and Epidemiology at http://www.epidem.com/). Information on the STROBE Initiative is available at www.strobe-statement.org.

For peer review only
